# Effect of Treatment of Clinical Seizures vs Electrographic Seizures in Full-Term and Near-Term Neonates: A Randomized Clinical Trial

**DOI:** 10.1001/jamanetworkopen.2021.39604

**Published:** 2021-12-17

**Authors:** Rod W. Hunt, Helen G. Liley, Deepika Wagh, Rachel Schembri, Katherine J. Lee, Andrew D. Shearman, Samantha Francis-Pester, Koert deWaal, Jeanie Y. L. Cheong, Monika Olischar, Nadia Badawi, Flora Y. Wong, David A. Osborn, Victor Samuel Rajadurai, Peter A. Dargaville, Bevan Headley, Ian Wright, Paul B. Colditz

**Affiliations:** 1 Department of Paediatrics, Monash University, Melbourne, Australia; 2 Clinical Sciences, Murdoch Children’s Research Institute, Melbourne, Australia; 3 Monash Newborn, Monash Children’s Hospital, Melbourne, Australia; 4 Cerebral Palsy Alliance, University of Sydney, Sydney, Australia; 5 Ritchie Centre, Hudson Institute of Medical Research, Monash University, Melbourne, Australia; 6 Mater Mother’s Hospital, Brisbane, Australia; 7 University of Queensland, Brisbane, Australia; 8 Perth Children’s Hospital, Perth, Australia; 9 Clinical Epidemiology Biostatistics Unit, Murdoch Children’s Research Institute, Melbourne, Australia; 10 Department of Paediatrics, University of Melbourne, Melbourne, Australia; 11 Department of Neonatal Medicine, John Hunter Children’s Hospital, Newcastle, Australia; 12 University of Newcastle, Callaghan, Australia; 13 Neonatal Services, The Royal Women’s Hospital, Melbourne, Australia; 14 Department of Obstetrics and Gynaecology, University of Melbourne, Melbourne, Australia; 15 University Children’s Hospital, Vienna, Austria; 16 Grace Newborn Intensive Care, The Children’s Hospital, Westmead, Australia; 17 Newborn Medicine, Royal Prince Alfred Hospital, Sydney, Australia; 18 University of Sydney, Sydney, Australia; 19 Department of Neonatology, KK Women’s and Children’s Hospital, Singapore; 20 Neonatal and Paediatric Intensive Care Unit, Royal Hobart Hospital, Hobart, Australia; 21 Menzies Institute for Medical Research, University of Tasmania, Hobart, Australia; 22 Department of Neonatal Medicine, Women’s and Children’s Hospital, Adelaide, Australia; 23 James Cook University, Cairns, Australia; 24 Royal Brisbane and Women’s Hospital, Brisbane, Australia

## Abstract

**Importance:**

Seizures in the neonatal period are associated with increased mortality and morbidity. Bedside amplitude-integrated electroencephalography (aEEG) has facilitated the detection of electrographic seizures; however, whether these seizures should be treated remains uncertain.

**Objective:**

To determine if the active management of electrographic and clinical seizures in encephalopathic term or near-term neonates improves survival free of severe disability at 2 years of age compared with only treating clinically detected seizures.

**Design, Setting, and Participants:**

This randomized clinical trial was conducted in tertiary newborn intensive care units recruited from 2012 to 2016 and followed up until 2 years of age. Participants included neonates with encephalopathy at 35 weeks’ gestation or more and younger than 48 hours old. Data analysis was completed in April 2021.

**Interventions:**

Randomization was to an electrographic seizure group (ESG) in which seizures detected on aEEG were treated in addition to clinical seizures or a clinical seizure group (CSG) in which only seizures detected clinically were treated.

**Main Outcomes and Measures:**

Primary outcome was death or severe disability at 2 years, defined as scores in any developmental domain more than 2 SD below the Australian mean assessed with Bayley Scales of Neonate and Toddler Development, 3rd ed (BSID-III), or the presence of cerebral palsy, blindness, or deafness. Secondary outcomes included magnetic resonance imaging brain injury score at 5 to 14 days, time to full suck feeds, and individual domain scores on BSID-III at 2 years.

**Results:**

Of 212 randomized neonates, the mean (SD) gestational age was 39.2 (1.7) weeks and 122 (58%) were male; 152 (72%) had moderate to severe hypoxic-ischemic encephalopathy (HIE) and 147 (84%) had electrographic seizures. A total of 86 neonates were included in the ESG group and 86 were included in the CSG group. Ten of 86 (9%) neonates in the ESG and 4 of 86 (4%) in the CSG died before the 2-year assessment. The odds of the primary outcome were not significantly different in the ESG group compared with the CSG group (ESG, 38 of 86 [44%] vs CSG, 27 of 86 [31%]; odds ratio [OR], 1.83; 95% CI, 0.96 to 3.49; *P* = .14). There was also no significant difference in those with HIE (OR, 1.77; 95% CI, 0.84 to 3.73; *P* = .26). There was evidence that cognitive outcomes were worse in the ESG (mean [SD] scores, ESG: 97.4 [17.7] vs CSG: 103.8 [17.3]; mean difference, −6.5 [95% CI, −1.2 to −11.8]; *P* = .01). There was little evidence of a difference in secondary outcomes, including time to suck feeds, seizure burden, or brain injury score.

**Conclusions and Relevance:**

Treating electrographic and clinical seizures with currently used anticonvulsants did not significantly reduce the rate of death or disability at 2 years in a heterogeneous group of neonates with seizures.

**Trial Registration:**

http://anzctr.org.au Identifier: ACTRN12611000327987

## Key Points

**Question:**

Does the treatment of all electrographic seizures in term or near-term neonates improve outcome at 2 years when compared with the treatment of clinical seizures alone?

**Findings:**

This randomized clinical trial analyzed 212 neonates at a high risk of seizures, 84% of whom had electrographic seizures. There was little evidence of a difference in either mortality or neurodevelopment impairment between the 2 groups.

**Meaning:**

These findings suggest that in full-term or near-term neonates with heterogeneous etiologies for seizures, treatment of electrographic seizures with conventional anticonvulsants was not associated with outcome.

## Teaser

This randomized control trial determines if the active management of electrographic and clinical seizures in encephalopathic term or near-term neonates improves survival free of severe disability at 2 years of age compared with only treating clinically detected seizures.

## Introduction

Seizures are the most common manifestation of neonatal encephalopathy, and with an estimated incidence of 1 to 5 per 1000 live births in term newborns, they are more common in the neonatal period than during any other time of life.^1,2,3^ The neurodevelopmental associations of neonatal seizures have been extensively described.^4^ They include motor and cognitive deficits,^5^ behavioral problems, such as attention deficit hyperactivity disorder and autism,^6^ and postneonatal epilepsy.^7^ While increased seizure burden is associated with increased risk of impaired neurodevelopment,^8^ there is evidence that even a single neonatal seizure can alter synaptic plasticity and have detrimental effects on cognitive functions, such as memory.^9^ The increasing awareness of the dangers of neonatal seizures has engendered a liberal approach to the use of anticonvulsants in the neonatal intensive care unit (NICU).

Most neonatal seizures are subclinical^10^; thus, bedside neuromonitoring with amplitude-integrated EEG (aEEG) has been widely adopted in NICUs^11,12^ to achieve recognition of most electrographic seizures^13^ and improve diagnostic precision.^14^ While the standard investigation for seizures in the NICU is still conventional EEG (cEEG), aEEG monitoring systems are suited to continuous use and bedside interpretation. In studies comparing cEEG with aEEG, aEEG has been shown to have sensitivity and specificity between 70% to 80% for detecting electrographic seizures.^15^

Anticonvulsant pharmacotherapy for neonatal seizures has changed little during the last 50 years.^16^ Almost all neonatologists still use phenobarbital as the first-line agent.^17,18^ Until 2020, the only published randomized controlled trial comparing anticonvulsants in neonates showed that phenobarbital was relatively ineffective as a first-line anticonvulsant, though similar to phenytoin, the other most popular drug used in the late 1990s.^19^ Rodent studies have shown that both phenobarbital and numerous other anticonvulsants can cause both apoptotic neurodegeneration^20^ and disrupted striatal synaptic development^21^ when administered during critical periods of brain development.

Clinicians are generally aware that most neonatal seizures are subclinical and that antiepileptic drugs are relatively ineffective and potentially harm the developing brain. Furthermore, the drugs and other disease-modifying treatments, such as therapeutic hypothermia, suppress clinical seizures more than electrical seizures. Electrical seizure detection using aEEG has, if anything, accentuated the dilemma—should all electrographic seizures, even without a clinical correlate, be treated to reduce the risk of subsequent impairment, or do the treatments themselves contribute to neurological disruption and injury?

When this study began, there was equipoise among participating centers regarding the balance of benefits and harms of identifying and treating electrographic seizures not associated with clinical signs to reduce overall seizure burden, inevitably increasing exposure to potentially neurotoxic drugs. The results of a similarly designed study^22^ in term neonates with hypoxic-ischemic encephalopathy (HIE) had shown a trend to reduction in seizure duration when both clinical and subclinical seizures were treated. However, the number of neonates enrolled in the study was small, and the study was underpowered to study neurodevelopmental outcome.

The objective of the current study was to determine if drug treatment of all clinically and electrographically detected seizures, compared with the treatment of clinically detected seizures alone, reduced mortality and neurodevelopmental morbidity through a potential reduction in seizure burden. We hypothesized that the treatment of all electrographic and clinical seizures would result in both a reduction in seizure burden and improved neurodevelopmental outcome.

## Methods

This randomized clinical trial was approved by the Human Research Ethics Committee at The Royal Children’s Hospital and registered with the Australian Clinical Trials Register (ACTRN12611000327987). Parents of eligible neonates provided written informed consent obtained by site investigators or trained delegates. This report follows the Consolidated Standards of Reporting Trials (CONSORT) reporting guideline for randomized clinical trials. The trial protocol is available in Supplement 1.

### Study Population

This prospective, randomized clinical trial examined neonates at risk of seizures to determine whether treatment of both clinical seizures and those evident from a visible aEEG monitor compared with the treatment of only clinically evident seizures (ie, masked monitor) reduced the rate of death and disability at 2 years of age. Neonates admitted to the NICU of a participating center were screened for eligibility between March 2012 and February 2016. Inclusion criteria were neonates more than 35 weeks’ gestational age (GA) who were younger than 48 hours of age, a diagnosis of either (1) neonatal encephalopathy including coma, stupor, or depressed mental state; (2) HIE with at least 2 of (i) Apgar score less than 5 at 5 minutes of birth, (ii) cord blood gas or arterial blood gas within 1 hour of birth with pH of less than 7.1 or base excess less than −12, or (iii) need for ongoing respiratory support at 10 minutes after birth; or (3) suspected neonatal seizures from any cause. It was anticipated that all neonates with HIE would receive therapeutic hypothermia as part of standard clinical care. Neonates were excluded if they had a diagnosis of nonconvulsive status epilepticus or cerebral dysgenesis was subsequently diagnosed on neuroimaging.

Recruitment occurred across 13 sites in 3 countries—Australia, Austria, and Singapore. Site investigators at each site participated in training for both the study and aEEG interpretation, based on a published aEEG interpretation guide,^23^ including criteria for diagnosing a seizure. Randomization was stratified by study site and diagnosis (ie, HIE or other), with group allocation being computer generated using block randomization with variable block sizes.

### Neuromonitoring and Neurodevelopmental Assessment

After randomization, the aEEG monitor was applied, and the screen was either left visible to the treating team so that both electrographic seizures and clinical seizures could be detected and treated for the clinical and electrographic seizure group (ESG) or covered so that only clinically detected seizures were treated for the clinical seizure group (CSG). The aEEG monitors were fitted with seizure detection software on all participants.^24^ Neither clinicians nor parents could be blinded to group allocation. In both arms of the study, clinically apparent seizures were treated. In the ESG, electrographic seizures fulfilling the diagnostic criteria were also treated if they lasted more than 2 minutes or occurred more than twice in 24 hours. We aimed to collect aEEG data for up to 5 days from randomization, but aEEG was removed where neonates recovered earlier and the aEEG was thought to interfere with bathing and other routine cares. Seizures were treated according to a pharmacological algorithm (eFigure in Supplement 2). Participants received routine newborn intensive care in all other respects. A Hammersmith Neonatal Neurological Examination (HNNE) was performed on day 7 of life, and a brain magnetic resonance imaging (MRI) was obtained between day 5 and day 14. At 2 years of age, participants were invited back to their recruiting site for a neurodevelopmental assessment using the Bayley Scales of Neonate Development, 3rd edition (BSID-III).^25^ Two-year assessments were performed by blinded outcome assessors. Information about vision, hearing, and presence or absence of cerebral palsy was obtained by medical assessment and history from the parents.

#### Data Collection

Demographic and perinatal clinical data collection, including anticonvulsant use, was recorded by site investigators. After recruitment, aEEGs were read blinded to group allocation and clinical details by 2 readers (RH and AS), and differences were resolved by discussion. A diagnosis of electrographic seizure was made when a rhythmic spike and wave pattern lasting at least 10 seconds with a definite beginning and end and associated with amplitude elevation visible on the aEEG compressed trace was detected. Seizure burden in seconds was calculated for each participant for as long as the aEEG monitor was attached. MRI brain scans were scored for damage using a published scoring system.^26^

The primary outcome was death or disability at 2 years of age, with a disability defined as any of: (1) BSID-III score greater than 2 SDs below the Australian population mean (ie, <78)^27,28^; (2) cerebral palsy (GMFCS>3); (3) visual acuity less than 6 of 60 in either eye; or (4) deafness requiring amplification. Participants who identified as meeting criteria for 1 component were classified as being disabled, with participants needing to have data on all components to be classified as not being disabled. Secondary outcomes were the components of the primary outcome, seizure burden in seconds, brain injury score from MRI at 5 to 14 days old, exposure to anticonvulsant in the perinatal period (in mg/kg for each anticonvulsant), time to full suck feeds, length of hospital stay, and postneonatal epilepsy in the first 2 years.

#### Sample Size

We estimated a background rate of death or neurodisability of 40% for our anticipated cohort, comprising approximately 50% of neonates with HIE as a cause for their encephalopathy. We based our sample size calculation on a 12% reduction in death or severe disability in the ESG. To achieve α = .05 and power of 80%, we aimed to have 260 neonates in each group. Allowing for 5% postrandomization exclusions for status epilepticus and cerebral dysgenesis and loss to follow-up of 10%, we aimed to recruit 300 neonates to each group. Recruitment commenced in March 2012 and ended prematurely at 212 neonates in January 2016 because of slow trial progress and a loss of equipoise at some sites with the publication of Srinivasakumar^29^ in October 2015 suggesting that treatment of all electrographic seizures was beneficial. The Trial Steering Committee decided the most prudent course of action was to redirect remaining resources to the collection of 2-year follow-up data and the analysis. Data analysis was completed in April 2021.

### Statistical Analysis

Analysis was by intention to treat. The primary outcome was summarized as the number and proportion in each group, with a comparison between the groups using logistic regression adjusted for site and diagnosis (HIE or other) as used in randomization, with results reported as an odds ratio (OR) and its 95% CI. In a sensitivity analysis, the analysis was repeated adjusting for the natural log of seizure burden (ie, allocating a seizure burden of 0.001 second to those with no seizures). An exploratory subgroup analysis in neonates with HIE was performed. Seizure burden was analyzed (in seconds) in 3 different ways—total seizure burden for the duration of the aEEG recording, seizure burden per day of total aEEG recording, and seizure burden from 12 to 72 hours after birth. These measures of seizure burden were compared between the groups using Poisson regression adjusted for site and diagnosis with results reported as incidence rate ratios with 95% CIs. Secondary outcomes were summarized by intervention group and compared between groups using logistic regression for dichotomous outcomes, and linear regression for continuous outcomes. Models were adjusted for site and diagnosis. For primary outcome and the binary language outcome, site was grouped into state within Australia sites and non-Australian sites, with no adjustment for site for the other binary outcomes because of the small numbers in some sites. Time to full suck feeds was summarized using medians and interquartile ranges (IQR) and analyzed using a Cox proportional hazards model adjusted for site and diagnosis. Analyses were conducted using the available data for each analysis in Stata version 15 (StataCorp). Statistical significance was set at *P* < .05, and tests were 2-tailed.

## Results

Between March 2012 and February 2016, 596 neonates were screened for inclusion, and 212 neonates were recruited, which totaled 106 neonates in each group. Of the 106 neonates in each group, 20 in each group were lost to follow-up or had incomplete data on the primary outcome, leaving 86 in each group available for the analysis of this outcome (Figure). The mean (SD) GA at birth was 39.2 (1.7) weeks and 122 (62%) were male for the 212 randomized participants. Demographic details were similar between the 2 groups (Table 1), with the exception that there were 50 girls (47%) in the ESG and 40 (38%) in the CSG. Of recruited neonates, 153 (72%) had a diagnosis of HIE (ie, all of whom received therapeutic hypothermia), with the other causes of seizures being arterial ischemic stroke (21 [10%]), extra-axial hemorrhage (16 [8%]), sinovenous thrombosis (3 [2%]), genetic epilepsies (3 [2%]), hypoglycemia (2 [1%]), and unknown (11 [5%]). No neonates were excluded from analysis with a diagnosis of cerebral dysgenesis.

**Figure.**
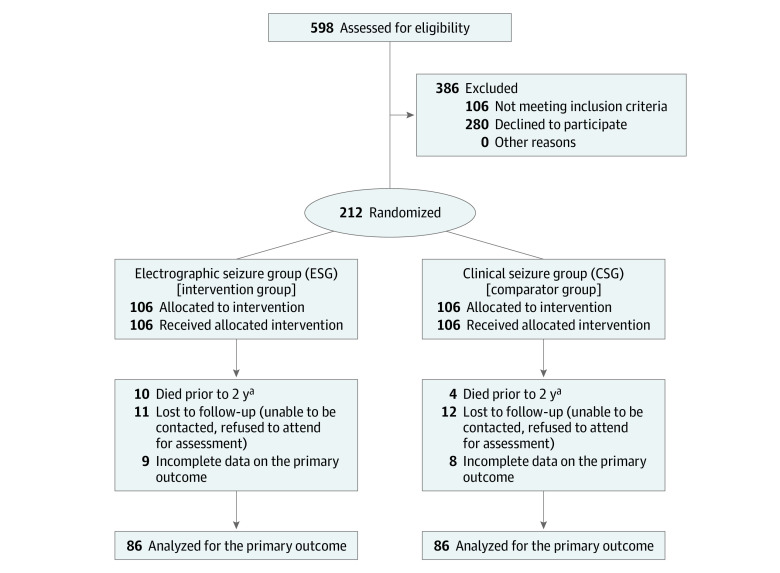
CONSORT 2010 Flow Diagram ^a^Included in the analysis of the primary outcomes.

**Table 1.** Demographics of Trial Participants

Demographics	No. (%)	
	ESG (n = 106)	CSG (n = 106)
Sex		
Female	50 (47%)	40 (38%)
Male	56 (53)	66 (62)
Gestation, mean (SD), weeks	39.2 (1.7)	39.2 (1.8)
Mode of delivery		
Vaginal		
Cephalic	32 (30.5)	23 (21.7)
Breech	1 (1.0)	2 (1.9)
Complex	2 (1.9)	3 (2.8)
Instrumental	20 (18.9)	28 (26.4)
Cesarean delivery		
Emergency	45 (42.9)	45 (42.5)
Elective	6 (5.7)	5 (4.7)
Diagnostic group		
HIE	78 (74)	74 (70)
Other	28 (26)	32 (30)
Resuscitation received at birth	78 (73.6)	96 (90.6)
Apgar Score, mean (SD), min		
1	2.7 (2.6)	2.9 (2.6)
5	4.6 (2.7)	4.9 (2.5)
Age at randomization, h	26.7 (13.4)	28.9 (13.7)
Site of recruitment		
Victoria, Australia	12	12
New South Wales, Australia	12	14
Queensland, Australia	48	47
Western Australia, Australia	25	27
Tasmania/South Australia, Australia	3	3
Non-Australia	6	3

Abbreviations: CSG, clinical seizure group; ESG, electrographic seizure group.

### Primary Outcome

The odds of death or disability were not significantly greater in the ESG group compared with the CSG group (n = 172; 38 of 86 [44%] vs 27 of 86 [31%]; odds ratio [OR], 1.83; 95% CI, 0.96-3.49; *P* = .14). The group difference was also not significant when adjusted for log seizure burden (n = 143; adjusted OR, 1.79; 95% CI, 0.87-3.67; *P* = .11). The group differences were not significant when the analysis was repeated in the subgroup of neonates with HIE both before (n = 128; OR, 1.77; 95% CI, 0.84-3.73; *P* = .14) and after (n = 101; adjusted OR, 1.48; 95% CI, 0.63-3.50; *P* = .52) adjustment for log seizure burden.

### Components of the Primary Outcome

The results for the components of the primary outcome are shown in Table 2. There was little evidence of differences between the 2 groups in any of the components. When scores from the BSID-III were analyzed as continuous variables, data were available for 162 neonates and there was evidence of lower cognitive scores for 80 survivors in the ESG compared with 82 in the CSG (mean [SD] scores, 97.4 [17.7] vs 103.8 [17.3]; mean difference, −6.5; 95% CI, −1.2 to −11.8; *P* = .02). A similar, although nonsignificant, trend was observed for the language scale, where data was available for 159 neonates, with reduced performance in the ESG (n = 77) compared with the CSG (n = 82) (mean [SD] score, 91.0 [20.7] vs 96.5 [19.5]; mean difference, −6.1; 95% CI, −0.2 to −12.4; *P* = .06).

**Table 2.** Summary of Components of the Primary Outcome

Components	Participants, No.	No. (%)		OR (95% CI)
		ESG	CSG	
Death	212	10 (9)	4 (4)	2.66 (0.81-8.78)
Cognitive disability	162	7 (9)	3 (4)	2.91 (0.70-12.1)
Motor disability	156	11 (14)	7 (9)	1.83 (0.66-5.08)
Language disability	159	22 (29)	13 (16)	2.26 (1.02-5.02)
Deafness	167	3 (4)	4 (5)	0.85 (0.18-3.98)
Blindness	170	3 (3.5)	6 (6.7)	0.53 (0.12-2.21)
Cerebral palsy	169	9 (11)	10 (12)	0.97 (0.37-2.54)

Abbreviations: CSG, clinical seizure group; ESG, electrographic seizure group; OR, odds ratio.

### Time to Suck Feeds

There was no evidence of a difference between groups in time to full suck feeds. In the ESG, the median (IQR) time to full feeds was 20.3 (11.3-40.3) days and 19.3 (10.6-40.2) days in the CSG (n = 200; hazard ratio, 0.98; 95% CI, 0.57-1.71; *P* = .97).

### Seizures

The duration of seizure burden and anticonvulsant use for all participants and the subgroup of participants with HIE is shown in Table 3. The aEEG data were available for 80 and 94 participants for the ESG and CSG, respectively, with missing data due to uninterpretable traces because of high impedance or interference from surrounding equipment in the NICU. In the ESG, 69 of 80 neonates (86%) and 65 of 94 neonates (69%) in the CSG were treated with anticonvulsant medication. In the ESG, 5 neonates (6%) and 4 neonates (4%) in the CSG developed postneonatal epilepsy, with little evidence of a difference between the 2 groups (n = 171; OR, 1.55; 95% CI, 0.39-6.14; *P* = .53).

**Table 3.** Seizure Burden and Anticonvulsant Use by Randomized Group

	No. (%)		IRR (95% CI)	HIE subgroup, IRR (95% CI)
	ESG	CSG		
Electrographic seizures present	69 (86)	78 (83)	OR, 1.28 (0.56 to 2.97)	OR, 1.68 (0.61 to 4.61)
Total seizure burden in total aEEG recording, median (IQR), s	848 (143 to 4840)	613 (60 to 3030)	1.61 (0.82 to 3.12)	1.12 (0.60 to 2.11)
Seizure burden, mean (SD), s				
Per day of total aEEG recording	325 (83 to 1355)	285 (86 to 1734)	1.19 (0.62 to 2.27)	0.85 (0.44 to 1.64)
From 12 to 72 h from birth	1063 (130 to 3725)	535 (90 to 3710)	0.91 (0.39 to 2.11)	0.63 (0.27 to 1.44)
Total phenobarbital dose, mean diff (95% CI), mg/kg	NA	NA	2.56 (−4.12 to 9.25)	5.31 (−3.67 to 14.3)
No.	69	64	NA	NA
Mean (SD)	38.2 (19.7)	34.6 (20.5)	NA	NA
Total phenytoin dose, mean difference (95% CI), mg/kg	NA	NA	−6.15 (−15.68 to 3.37)	−10.9 (−27.60 to 5.82)
No.	24	12	NA	NA
Mean (SD)	20.3 (10.0)	27.7 (20.7)	NA	NA
Medications administered, No.			NA	NA
1	43	49	NA	NA
2	10	6	NA	NA
3	12	7	NA	NA
≥4	4	3	NA	NA

Abbreviations: aEEG, amplitude integrated electroencephalography; CSG, clinical seizure group; ESG, electrographic seizure group; HIE, hypoxic-ischemic encephalopathy; IRR, incident rate ratio; NA, not applicable; OR, odds ratio.

### MRI

There was little evidence that MRI brain injury scores were different between the ESG and CSG (n = 181; mean [SD] score, 10.18 [10.15] vs 9.37 [8.6]; mean difference, 0.96; 95% CI, −1.82 to 3.74; *P* = .50). There was little evidence of difference between groups in gray matter, white matter, or cerebellum subscores.

## Discussion

We report results from the largest randomized controlled trial to date comparing 2 approaches to the pharmacological management of neonatal seizures. In the first approach, both clinical and electrographic seizures detected by aEEG were treated, and in the second approach, only clinical seizures were treated while an aEEG monitor was masked. In a heterogeneous group of neonates with seizures or suspected of having seizures, we found no significant difference in odds of death or disability at 2 years of age between the ESG group and the CSG group, possibly because the study was underpowered to detect such a difference. In addition, there was evidence that survivors at 2 years in the CSG had better cognitive scores than those in the ESG, but not language scores or motor outcome. Of note, only 84% of our recruited sample had electrographic seizures, underscoring that prediction and diagnosis of neonatal seizures is difficult, and even with access to aEEG monitoring, clinicians may tend to overdiagnose.^30^ In the ESG compared with the CSG, there were slightly more girls, and fewer neonates required resuscitation at birth. This could have potentially introduced bias in the treatment effect estimate, causing the ESG to perform better than the CSG on measures of neurodevelopment. However, where there was a trend to difference, it occurred in the other direction.

Our findings were not dissimilar to those of the 2 previous studies performed with a similar study design.^22,29^ Van Rooij et al^22^ randomized 42 neonates, and in 11 survivors, who had only clinical seizures treated, they found evidence of a correlation between duration of seizures and MRI brain injury score. However, the median MRI score was 4 in both CSG and ESG. Like our study, recruitment to a trial involving the concealment of neuromonitoring data at a time when parents were under extreme stress and anxiety, was difficult, and recruitment fell well short of the target sample size. Srinivasakumar et al^29^ randomized 72 neonates and had outcomes at 2 years for 61 neonates. Their methods primarily differed in the use of conventional EEG on all participants to determine if seizures detected on aEEG were seizures warranting treatment. Only 35 of 72 neonates had confirmed electrographic seizures. They reported a lower seizure burden in their ESG than the CSG, although they found no difference between the groups in BSID-III scores at 18 to 24 months. While there was no difference in primary outcome on intention to treat analysis, they found a reduction in scores across all domains on the BSID-III with increasing seizure burden when their entire cohort was analyzed. They concluded that targeted treatment of electrographic seizures in neonates with HIE could improve outcomes. The results of this trial resulted in a loss of equipoise at some sites in relation to this clinical question, and many sites now continue to treat all electrographic seizures.

In addition to the reduced sample size, the lack of evidence for a difference between groups in the current study may be explained by the heterogeneous nature of our patient population. With the rapidly expanding use of aEEG in Australian NICUs, we deliberately designed a pragmatic trial targeting all neonates with seizures or at risk of having neonatal seizures. Our cohort contained a small number of neonates with extra-axial hemorrhage due to birth trauma, who had seizures but for whom a favorable neurodevelopmental outcome was likely. We also included neonates with arterial ischemic stroke, genetic epilepsies, and intracerebral hemorrhage secondary to sinovenous thrombosis or coagulopathy, for whom cerebral injury, and therefore prognosis, may have been less modifiable by seizure burden or anticonvulsants. Nevertheless, subgroup analysis including only those neonates with HIE also showed weaker evidence of a difference between the groups concerning to the primary outcome.

The 5% rate of postneonatal epilepsy in our cohort was also much lower than previously reported.^31^ This outcome was collected by parent report on assessment at 2 years and may underestimate the true rate. In addition, this finding may reflect both the heterogeneous nature of our cohort and the difficulties inherent in diagnosing epilepsy at a young age.

The major strength of the current study is that it is the largest randomized clinical trial to assess the use of treating electrographic and clinical seizures in the first 48 hours of life to date, with external validity improved by the multicenter recruitment and the pragmatic approach of enrolling all neonates at high risk of seizures, not just those with HIE.

### Limitations

Our study was limited. With the early closure of recruitment related to feasibility and loss of equipoise, the study was underpowered concerning the primary and secondary outcomes. We did not include cEEG verification of aEEG findings, mainly because it is usually not available in the Australian perinatal context. At the time this trial commenced, there was perhaps an optimistic reliance on the diagnostic capability of aEEG. However, in retrospect, we agree with others that conventional EEG remains the reference standard for the detection of neonatal seizures,^32^ and essential in the validation of neonatal research.^33^ Availability of cEEG in NICUs is increasing, but even with this technology, the timely and appropriate targeting of treatment for neonatal seizures will remain challenging.^34^

Despite our increasing reliance on bedside aEEG as a neuromonitoring tool, the issue of whether or not seizure detection should trigger administration of conventional, potentially neurotoxic, anticonvulsant drugs remains unresolved. The evidence for seizures exacerbating cerebral damage is increasing, and as such the search for more effective anticonvulsant therapy should be a focus of future research.

## Conclusions

In this randomized control trial, there was little evidence of difference in mortality or morbidity at 2 years of age between an ESG and CSG; however, our study was underpowered. Contrary to what we hypothesized, we report an association of improved cognitive outcomes at 2 years in the CSG.

## Notes

Supplement 1.
Trial Protocol and Statistical Analysis Plan

Supplement 2. **eFigure.** Treatment Algorithm for Seizures

Supplement 3.
Group Members

Supplement 4.
Data Sharing Statement
