# Supplementary material for: Effect of Treatment of Clinical Seizures vs Electrographic Seizures in Full-Term and Near-Term Neonates: A Randomized Clinical Trial
Source: JAMA Netw Open. 2021 Dec 17;4(12):e2139604. doi: 10.1001/jamanetworkopen.2021.39604 (PMC8683963; doi:10.1001/jamanetworkopen.2021.39604)
Supplement: Supplement 1. — Trial Protocol and Statistical Analysis Plan [file jamanetwopen-e2139604-s001.pdf]

Version 3.0	# Statistical Analysis Plan

Neonatal Electrographic Seizure Trial (NEST)
1006053

A randomized controlled trial comparing the treatment of electrographic and clinical seizures, to the treatment of clinical seizures alone, in term or near-term encephalopathic infants and measuring the impact on death and neurodevelopment at 2 years

Document Version History

Version Date	Version	Author	Signature	Change Description	Reason/Comment
08-08-2018	1.0	Rachel Schembri		Initial release	To be reviewed by RH and KL.
09-05.2019	2.0	Rachel Schembri and Rod Hunt		For initial data analysis	RV by KL
26.07.2021	3.0	Rod Hunt		For journal submission	Tidy up

TABLE OF CONTENTS

LIST OF ABBREVIATIONS 3

1. STUDY OBJECTIVES 4
1.1. OVERVIEW 4
1.2. PRIMARY OBJECTIVE 4
1.3. SECONDARY OBJECTIVES 4

2. BACKGROUND/INTRODUCTION 4
2.1. STUDY DESIGN 4
2.2. TREATMENT GROUPS 4
2.3. STUDY POPULATION 5
2.4. INTERVENTION 5
2.5. SAMPLE SIZE 5
2.6. STUDY PROCEDURE 5

3. POPULATIONS OF ANALYSIS 6

4. OUTCOME VARIABLES 6
4.1. PRIMARY OUTCOME 6
4.2. SECONDARY PARAMETERS OUTCOMES 7
4.3. OTHER PARAMETERS 8

5. STATISTICAL METHODOLOGY 9
5.1. GENERAL METHODOLOGY 9

LIST OF ABBREVIATIONS

AE	Adverse Event
aEEG	Amplitude-integrated Electroencephalography
BP	Blood Pressure
BSID-III	Bayley Scales of Infant Development, 3rd Edition
CI	Confidence Interval
CRF	Case Report Form
GCP	Good Clinical Practice
ITT	Intent-To-Treat
NICU	Newborn Intensive Care Unit
SAE	Serious Adverse Event
SD	Standard Deviation
SE	Standard Error
MedDRA	Medical Dictionary for Regulatory Activities
WHO DD	World Health Organization Drug Dictionary

1. STUDY OBJECTIVES

1.1. OVERVIEW

Neonatal seizures are an important sign of neurological disease, and there is evidence emerging that neonatal seizures may cause harm. It is also acknowledged that clinical seizures in newborn infants are difficult to recognise and that 'sub-clinical' electrographic seizures may have the same potential for harm, even without a clinical correlate. Many NICU's are now investing in bedside aEEG monitoring although the evidence for improved clinical outcome following use of such monitoring is limited. This multi-centre randomized controlled trial will therefore address the question of whether or not treating electrographic seizures detected by aEEG results in improved neuro-developmental outcome at two-years of age.

1.2. PRIMARY OBJECTIVE

The primary study objective is to determine if the active management of both electrographic and clinical seizures in encephalopathic term or near-term infants improves mortality and severe disability at two-years of age, compared to when only clinically detected seizures are treated.

1.3. SECONDARY OBJECTIVES

1. To compare the effect of treatment of EEG seizures detected on aEEG monitoring, on brain injury assessed by cerebral magnetic resonance imaging (MRI) performed at day 5 to 14 compared to when only clinically detected seizures are treated.
2. To explore correlations between seizure burden and the extent of cerebral injury assessed by MRI at day 5 to 14 in term or near term encephalopathic infants.
3. To compare clinically meaningful short term outcomes such as time to full suck feeds and length of hospital stay between the two groups.

2. BACKGROUND/INTRODUCTION

2.1. STUDY DESIGN

The study was a prospective randomized, parallel controlled trial, conducted across 13 national and international centres. Patients were randomized to one of two treatment groups: intervention or (active) control.

The intervention group received active treatment for both electrographic and clinical seizures, while the control group received treatment for clinical seizures only, as per standard neonatal care. The study was hospital-based and recruited current inpatient participants. The follow-up at 2 years was conducted with participants as outpatients. The study was designed to determine superiority of active treatment of both electrographic and clinical seizures, compared with treatment of clinical seizures alone.

2.2. TREATMENT GROUPS

Patients were randomized to one of two treatment groups: intervention or control. Randomisation was stratified by site and diagnosis (Hypoxic-Ischemic encephalopathy or 'other') and used variable block size. Randomisation was accessed by centres via a web-based platform (with opaque envelopes in place as a back up).

It was not possible to fully blind this study due to the nature of the intervention. Treating doctors and nurses knew which infants were randomised to intervention and control arms. Assessment of

the primary outcome was carried out by a psychologist who was not involved with the family or subject at the time of recruitment and acute illness. Parents were asked not to divulge which arm of the study their infant was in (where this is known by them). The psychologist assessing the primary outcome will thus be blinded.

2.3. STUDY POPULATION

Infants ≥ 35 weeks' gestation (term or near term) at birth admitted to participating neonatal intensive care units who satisfied the following criteria were approached for study participation. Participants were required to be ≤ 48 hours old at the time of randomization, and have a diagnosis of either:

- Neonatal encephalopathy including coma, stupor, or depressed mental state (based on modified Sarnat classification II-III33)
- Hypoxic-ischemic encephalopathy or at risk for hypoxic-ischemic encephalopathy (two of: Apgar score < 5 at 5 minutes; Cord blood gas or postnatal blood gas within 1 hour of birth with pH <7.1 or BE > –12 within one hour of birth; Need for ongoing respiratory support at 10 minutes after birth)
- Suspected neonatal seizures with an assessment of whether the infant is truly having seizures being made on arrival at the NICU by the treating physician. If the infant is thought to have had seizures, they will be deemed eligible for the trial.

Potential participants were excluded if < 35 completed weeks of gestation at birth, or would be more than 48 hours old at the time of randomization. A diagnosis of non-convulsive status epilepticus or cerebral dysgenesis also precluded participation.

2.4. INTERVENTION

Amplitude-integrated EEG (aEEG) was recorded for all participants. Participants who were randomised to intervention had aEEG in use clinically for the detection of seizures in real time, and both electrographic and clinical seizures were actively treated. Participants randomized to the control group did not have aEEG in use clinically (signals were recorded but screens were covered and not observed for clinical use) and treatment was administered for clinical seizures only.

2.5. SAMPLE SIZE

Power calculations were based on finding a 12% reduction in death or severe disability at 2 years, based on an assumed rate of 40% in the control group, which would be an extremely important difference in practice. Recruitment of 260 infants in each group would be required to detect a 12% reduction in death or severe disability at 2 years (alpha value of 0.05 and power of 80%). Some infants initially eligible will be excluded post-randomisation because ineligibilities will be identified during the study. Allowing for a post-randomisation exclusion rate of 5% plus a loss to follow-up rate of 10%, 299 infants were planned to be recruited to each group.

Actual recruitment, underpowered. Recruitment to this trial was terminated following a recommendation from the DSMC in late 2015 because of (a) loss of equipoise secondary to publication by the St Louis group (Pediatrics 2015; 136(5)), and (b) slow recruitment and recognition that completion of recruitment in reasonable time frame unlikely.

2.6. STUDY PROCEDURE

Eligible infants were identified on admission to the newborn intensive care unit at one of the participating sites. Once admitted, parents of eligible infants were approached to determine if they would consider participation in a research study, and if so informed consent was sought.

Following consent, conventional EEG was performed for all participants as soon as practicable following enrolment to confirm participant was not in status epilepticus. Baseline demographic information, neurological examination (Sarnat stage) and medical history were also collated at this time. Once eligibility was confirmed, randomization was conducted (≤ 48 hours after birth) and the aEEG monitor attached for a period of up to 7 days (or until clinical improvement allows the infant to be transferred out of intensive care). 'Seizure burden' and 'anticonvulsive drug' use was recorded throughout this time and through to discharge. Adverse events were also monitored throughout.

In the window of day 5 through 14, a cerebral MRI was conducted. This time frame was deliberate to allow different centres to obtain imaging within this time period. Infants were be fed, wrapped in a vacu-fix® beanbag and imaged whilst asleep. Sequences obtained included conventional T1- and T2-weighted imaging, as well as diffusion weighted imaging and magnetic resonance spectroscopy. The exact parameters for these sequences will vary from centre to centre depending on the make and strength of the magnet.

On Day 7 (or prior if discharge is prior to this), the aEEG monitor was removed and the Dubowitz neurobehavioural assessment was conducted. At discharge from hospital, 'time to full oral feeds' and 'length of hospital stay' were recorded.

Once the infant reached 2 years of age, a 2 hour outpatient assessment was conducted. The Bayley Scales of Infant Development 3rd Edition (BSID-III) was carried out to assess developmental outcomes via developmental play tasks. At this time, post-neonatal epilepsy rate and anticonvulsive drug use were also documented.

3. POPULATIONS OF ANALYSIS

Outcome data will be analysed using the intention to treat principle. All participants will be analysed in their randomized group, regardless of any crossover or discontinuation of aEEG recording. Participants withdrawn due to *a priori* ineligibility criteria at any stage during the study will not be included as per the original study protocol.

4. OUTCOME VARIABLES

4.1. PRIMARY OUTCOME

The primary outcome is death or neurodisability at 2 years of age. This will be summarised as the number and proportion in each intervention group.

Neurodisability will be measured by the BSID-III. Moderate to severe neurodisability is defined as a composite score of less than 78 (2 standard deviations below the Australian mean; mean=108, SD=15). A child will be considered to have neurodisability if they score less than 78 on any one of the BSID: Cognitive composite score, Language composite score, Motor composite score. A child will also be classified as having neurodisability if they have CP or epilepsy, or are deaf or blind, measured at 2 years of age.

MEASURE	TYPE OF VARIABLE	DESCRIPTION
**Death**		
	Dichotomous	Derived from 'Date of death', 'Time of death' 'Reason for death' and adverse events.

**Neurodisability (BSID-III)**	Dichotomous	Disability classified if any of the below are present:
Cognitive composite score <78	Continuous	0-160 where higher scores represent better cognition.
Language composite score <78	Continuous	0-160 where higher scores represent better language.
Motor composite score <78	Continuous	0-160 where higher scores represent better motor control.
CP present		Diagnosed at 2 yrs
Epilepsy present	Dichotomous	Development of epilepsy by age two, derived from 'Current documented diagnosis of epilepsy'.
Child deaf		2 years
Child blind		2 years

4.2. SECONDARY PARAMETERS OUTCOMES

<<Describe the secondary outcome variables, including parameter categorization (if applicable)>>

2 year old outcomes (detailed): cognitive, language, motor, CP, deaf, blind, epilepsy

MEASURE	TYPE OF VARIABLE	DESCRIPTION
**MRI**		
Grey matter	Ordinal	0=No abnormality, 1=Some abnormality, 2=Extensive abnormality
White matter / cortex	Ordinal	0=No abnormality, 1=Some abnormality, 2=Extensive abnormality
Cerebellum	Ordinal	0=No abnormality, 1=Some abnormality, 2=Extensive abnormality
IVH	Ordinal	0=No abnormality, 1=Some abnormality, 2=Extensive abnormality
SDH	Ordinal	0=No abnormality, 1=Some abnormality, 2=Extensive abnormality
CSVT	Ordinal	0=No abnormality, 1=Some abnormality, 2=Extensive abnormality
TOTAL SCORE	Continuous	Sum of above 6 scores (0-12)
**Seizure burden**		
Seizure time	Continuous	Minutes spent in seizure (in seconds on original scale, to be transformed)
Number of seizure events	Continuous	Number of seizures
Average number of seizure events per hour of recording		Derived from recording time and number of seizure events
Average time spent in seizure per hour of recording		Derived from recording time and seizure time
Duration of shortest seizure	Continuous	Seconds
Duration of longest	Continuous	Seconds

seizure		
Count of babies who had seizures	Continuous	Count
**Anticonvulsant use**		
Number of anticonvulsants	Continuous	Number of anticonvulsant drugs used during inpatient stay, and at 2 year follow-up.
Anticonvulsant dose	Continuous	Cumulative dose of anticonvulsants expressed in mg/kg for each anticonvulsant used.

OTHER MEASURES REQUIRED FOR DESCRIPTIVES OR OUTCOMES

**MEASURE**	**TYPE OF VARIABLE**	**DESCRIPTION**
**MRI**		
Count of babies who had MRI	Continuous	Count
Age at time of MRI		Youngest, oldest and median
**Seizure burden**		
aEEG recording time	Continuous	Minutes of recording, derived from dates and times recording started and ended
Age at time of aEEG start?	Continuous	
**Anticonvulsant use**		
Number of anticonvulsants	Continuous	Number of anticonvulsant drugs used until discharge
Anticonvulsant dose	Continuous	Cumulative dose of anticonvulsants expressed in mg/kg for each anticonvulsant used.
Discharged on anticonvulsant	dichotomous	
**Other**		
HIE diagnosis	Nominal	Babies diagnosed with birth asphyxia
Time to full suck feeds	Continuous	Hours and minutes from birth.
Failure to achieve full suck feeds prior to discharge	dichotomous	
Time to discharge from hospital	Continuous	Hours and minutes from birth.
Development of epilepsy by age two	Dichotomous	Derived from 'Current documented diagnosis of epilepsy'.

4.3. OTHER PARAMETERS

DEMOGRAPHY AND BASELINE

Birthweight, Birthweight z-score, Gestational age at birth, gender, Apgar scores

SAFETY

Number of AE's and SAE's until the time of discharge.

5. STATISTICAL METHODOLOGY

5.1. GENERAL METHODOLOGY

Rachel to complete…for SAP and manuscript

PRIMARY ANALYSIS

Randomisation will be stratified by site (13) and diagnosis (HIE or other) – regression with these as covariates rather than chi square (above) and t-tests (below). All results will be presented as unadjusted comparisons and adjusted for site and diagnosis (stratification factors), and whether the infant was cooled (for HIE infants) using linear (continuous outcomes) and logistic (binary outcomes) regression.

Neurodisability = cognitive, language, motor (mean 108 and SD 15, anything below 2SDs is moderate to severe disability) or CP. Categorical for primary outcome, number with death or neurodisability on ANY of these versus alive and NONE. Descriptive also (continuous by group, and number in each SD bracket, by group).

Analysis will be by intention to treat. The primary outcome will be summarised as the number and proportion in each group, with a comparison between the groups using logistic regression adjusted for site and diagnosis (HIE/other) as used in randomisation, with results reported as an odds ratio (OR) and its 95% confidence interval (CI).

SECONDARY ANALYSES

Secondary outcomes will be summarised by intervention group, and compared between groups using logistic regression for dichotomous outcomes, and linear regression for continuous outcomes. Models will be adjusted for site and diagnosis. Time to full suck feeds will be summarised using medians and interquartile ranges (IQR) and analysed using a Cox proportional hazards model adjusted for site and diagnosis. Analyses will be conducted using the available data for each analysis in Stata v15.

All results will be presented as unadjusted comparisons and adjusted for site and diagnosis (stratification factors), and whether the infant was cooled (for HIE infants) using linear (continuous outcomes) and logistic (binary outcomes) regression.

- Does MRI (components or total) correlate with seizure burden?
- Does MRI (components or total) correlate with 2 year outcome?
- If yes, is this correlation stronger for the HIE group than the whole cohort?
- Does MRI (components or total) change the association between phenobarbitone exposure and cognitive outcome (already adjusted for seizure burden)
-

HANDLING OF MISSING DATA

aEEG data used if subject has more than 24 hours of aEEG data

SENSITIVITY ANALYSES

In a sensitivity analysis, the analysis for primary outcome will be repeated adjusting for the natural log of seizure burden (allocating those with no seizures a seizure burden of 0.001 second). Seizure burden will be analysed (in seconds) in three different ways (a) total seizure burden for the duration of the aEEG recording (b) seizure burden per day of total aEEG recording and (c) seizure burden from 12 to 72 hours from birth. These measures of seizure burden will be compared between the groups using Poisson regression adjusted for site and diagnosis, with results reported as incidence rate ratios along with 95% CIs.

SUBGROUP ANALYSIS

All primary and secondary analyses will be repeated in the subgroup of participants with a HIE diagnosis.

CLASSIFICATION OF PROTOCOL VIOLATION

Protocol violations will be summarized as the number of violations and the proportion of participants with a violation by treatment arm.

1006053

A randomized controlled trial comparing the treatment of electrographic and clinical seizures, to the treatment of clinical seizures alone, in term or near-term encephalopathic infants and measuring the impact on death and neurodevelopment at 2 years

Neonatal Electrographic Seizure Trial (NEST)

Version 6: 17 June 2014

CONFIDENTIAL

This document is confidential and the property of Murdoch Childrens Research Institute.

No part of it may be transmitted, reproduced, published, or used without prior written authorization from the institution.

STATEMENT OF COMPLIANCE

This document is a protocol for a clinical research study. The study will be conducted in compliance with all stipulations of this protocol, the conditions of ethics committee approval, the NHMRC National Statement on Ethical Conduct in Human Research (2007) and the Note for Guidance on Good Clinical Practice (CPMP/ICH-135/95).

© 2021 Hunt RW et al. *JAMA Network Open*.

TABLE OF CONTENTS

		Page
	INVESTIGATORS AND FACILITIES	
1.1	Study Location/s	5
1.2	Study Management	6
	1.2.1 Principal Investigator	6
	1.2.2 Statistician	7
	1.2.3 Internal Trial Committees	8
	1.2.4 Independent Safety and Data Monitoring Committee	8
1.3	Funding and resources	8
1.	INTRODUCTION AND BACKGROUND	
2.1	Background Information	8
2.2	Research Question	10
2.3	Rationale for Current Study	10
2.	STUDY OBJECTIVES	
3.1	Primary Objective	10
3.2	Secondary Objectives	11
4.	STUDY DESIGN	
4.1	Type of Study	11
4.2	Study Design Diagram	11
4.3	Number of Subjects	12
4.4	Expected Duration of Study	12
4.5	Primary and Secondary Outcome Measures	12
5.	STUDY TREATMENTS	
5.1	Treatment Arms	12
	5.1.1 Description	12
5.2	Measurement of subject compliance	12
5.3	Excluded medications and treatments	13
6.	SUBJECT ENROLLMENT AND RANDOMISATION	
6.1	Recruitment	13
6.2	Eligibility Criteria	13
	6.2.1 Inclusion Criteria	13
	6.2.2 Exclusion Criteria	14
6.3	Randomisation Procedures	14
6.4	Blinding Arrangements	14
6.5	Subject Withdrawal	14
	6.5.1 Reasons for withdrawal	14
	6.5.2 Handling of withdrawals and losses to follow-up	15
	6.5.3 Replacements	15
6.6	Trial Closure	15
7.	STUDY VISIT AND PROCEDURE SCHEDULE	16
8.	CLINICAL AND LABORATORY ASSESSMENTS	17
9.	ADVERSE EVENT REPORTING	
9.1	Definitions	18
9.2	Assessment and Documentation of Adverse Events	19

© 2021 Hunt RW et al. *JAMA Network Open*.

9.3 Eliciting Adverse Event Information 20
9.4 Serious Adverse Event Reporting 20
9.4.1 SAEs 20
9.4.2 SUSARs 21
10. STATISTICAL METHODS ....
10.1 Sample Size Estimation 21
10.2 Population to be analysed 21
10.3 Statistical Analysis Plan 22
10.4 Interim Analyses 22
11. DATA MANAGEMENT ....
11.1 Data Collection 22
11.2 Data Storage 23
11.3 Study Record Retention 23
12. ADMINISTRATIVE ASPECTS ....
12.1 Confidentiality 23
12.2 Independent HREC Approval 24
12.3 Modifications of the protocol 24
12.4 Protocol Deviations 24
12.5 Participant Reimbursement 24
12.6 Financial Disclosure and Conflicts of Interest 24
13. USE OF DATA AND PUBLICATIONS POLICY 25
14. REFERENCES 26
15. APPENDICES ....
Appendix 1- Treatment algorithm.. 28
Appendix 2 – Trial Steering Committee – Terms of Reference......... 29
Appendix 3 – Data Monitoring Committee – Terms of Reference..... 33
Appendix 4 – MRI Scoring System... 37

© 2021 Hunt RW et al. *JAMA Network Open*.

PROTOCOL SYNOPSIS

Title	Neonatal Electrographic Seizure Trial (NEST)
Objectives	To determine if the active management of electrographic seizures in encephalopathic term born infants improves neurodevelopmental outcome.
Study design	A multi-centre, randomized, controlled , 2-arm parallel study
Outcomes	1. All cause mortality<br>2. Severe disability defined as motor and/or cognitive delay more than 2 standard deviations below the mean for all recruited subjects.
Study Duration	5 years – including 3 years active recruitment, 2 years follow up and 6 months data analysis.
Number of Subjects	630 infants
Population	Infants at least 35 weeks gestation and no more than 48 hours old admitted to a participating Neonatal Intensive Care Unit (NICU) with a diagnosis of neonatal encephalopathy, hypoxic-ischemic encephalopathy or suspected neonatal seizures.

GLOSSARY OF ABBREVIATIONS

**ABBREVIATION**	**TERM**
aEEG	Amplitude-integrated electroencephalography
AE	Adverse Event
CEBU	Clinical Epidemiology and Biostatistics Unit
cEEG	Conventional Electroencephalography
CSG	Clinical Seizure Group
CP	Cerebral Palsy
CPI	Coordinating Principal Investigator
CRF	Case Report Form
DSMC	Data and Safety Monitoring Committee
DW-MRI	Diffusion-weighted Magnetic Resonance Imaging
ESG	Electrographic Seizure Group
HIE	Hypoxic-ischemic encephalopathy
HREC	Human Research Ethics Committee
Kg	Kilogram
MRS	Magnetic Resonance Spectroscopy
Mg	Milligram
NICU	Neonatal Intensive Care Unit
PI	Principal Investigator
RCH	Royal Children's Hospital
SAE	Serious Adverse Event
SID	Study Identification Number
SC	Study Coordinator
SUSAR	Serious Unexpected Suspected Adverse Event
Sz	Seizure
TSC	Trial Steering Committee

© 2021 Hunt RW et al. *JAMA Network Open*.

1. INVESTIGATORS AND FACILITIES

1.1 Study Location/s

**Coordinating Centre**<br>Department of Neonatology<br>The Royal Childrens Hospital<br>50 Flemington Road<br>PARKVILLE Victoria 3052	Neonatal Intensive Care and Special Care Nursery<br>The Royal women's Hospital<br>20 Flemington Road<br>PARKVILLE Victoria 3052
Grace Centre for Newborn Care<br>The Children's Hospital at Westmead<br>Cnr Hawkesbury Rd and Hainsworth St<br>WESTMEAD New South Wales 2145	Monash Newborn<br>Monash Medical Centre<br>246 Clayton Road<br>CLAYTON Victoria 3168
Mater Neonatal Services<br>Mater Mothers' Hospital<br>Stanley Street<br>SOUTH BRISBANE Queensland 4101	Grantley Stable Neonatal Unit<br>Royal Brisbane and Women's Hospital<br>Level 5, Ned Hanlon Building<br>Butterfield Street<br>HERSTON Queensland 4029
Neonatalogy Clinical Care Unit (NCCU)<br>King Edward Memorial Hospital<br>1st Floor, Block A<br>374 Bagot Road<br>SUBIACO Western Australia 6008	Neonatalogy Clinical Care Unit<br>Princess Margaret Hospital<br>Roberts Road<br>SUBIACO Western Australia 6009
Neonatal Intensive Care Unit<br>John Hunter Children's Hospital<br>Lookout Road<br>NEW LAMBTON New South Wales 2310	Department of Neonatal Medicine<br>Women's and Children's Hospital<br>72 King William Road<br>NORTH ADELAIDE South Australia 5006
Department of Neonatal Medicine<br>Royal Prince Alfred Newborn Care<br>Royal Prince Alfred Hospital<br>Missenden Road<br>CAMPERDOWN New South Wales 2050	Neonatal Intensive Care Unit<br>Royal Hobart Hospital<br>48 Liverpool Street<br>HOBART Tasmania 7000
Special Care Nursery<br>Cairns Hospital<br>165 The Esplanade<br>CAIRNS Queensland 4870	Neonatal Unit<br>Flinders Medical Centre<br>Flinders Drive<br>Bedford Park South Australia 5042
KK Women's and Children's Hospital<br>100 Bukit Timah Road<br>SINGAPORE 229899	University Children's Hospital Vienna<br>Währinger Gürtel 18-20<br>1090 Vienna Austria

© 2021 Hunt RW et al. *JAMA Network Open*.

1.2 Study Management

The Coordinating Principal Investigator (CPI) and the study coordinator (SC) located at The Royal Childrens Hospital Melbourne will be responsible for the overall conduct of the study including site initiation, data entry and database maintenance, monitoring and statistical analysis. The study team will consist of principle investigators and research nurses located at individual participating sites.

Principal investigators (PI) will be responsible for the overall study conduct at their site/s, including delegation of the informed consent process, clinical assessment and medical management of study participants. Research nurses may be delegated the responsibility of informed consent, coordinating participant follow up visits, data collection and maintenance of study documentation. The study statistician will be responsible for data cleaning and analysis.

1.2.1 Principal Investigator

Coordinating Principal Investigator<br>Dr Rod Hunt, FRACP, PhD<br>Director, Department of Neonatal Medicine<br>The Royal Children's Hospital<br>50 Flemington Road<br>PARKVILLE Victoria 3052<br>Tel: +61 3 9345 5008<br>Fax: +61 3 9345 5067<br>E-mail: rod.hunt@rch.org.au	Dr Jeanie Cheong<br>Consultant Neonatologist<br>The Royal Women's Hospital<br>Locked Bag 300<br>PARKVILLE Victoria 3052<br>Tel: +61 3 8345 3771<br>Fax: + 61 3 8345 3789<br>E: jeanie.cheong@thewomens.com.au
Professor Nadia Badawi<br>Director, Grace Newborn Nurseries<br>Children's Hospital Westmead<br>Locked Bag 4001<br>WESTMEAD New South Wales 2145<br>Tel: +61 2 9845 2715<br>Fax: +61 2 9845 2251<br>E: nadiab@chw.edu.au	Dr Flora Wong<br>Consultant Neonatologist<br>Level 5, 246 Clayton Road<br>CLAYTON Victoria 3168<br>Tel: +61 3 9594 5482<br>E: flora.wong@monash.edu
Dr Helen Liley<br>Senior Staff Specialist, Neonatology<br>Mater Health Services<br>Kevin Ryan Centre<br>Raymond Terrace<br>SOUTH BRISBANE Queensland 4101<br>T: +61 7 3163 2733<br>F: + 61 7 3163 1435<br>E: Helen.Liley@mater.org.au	Professor Paul Colditz<br>Grantley Stable Neonatal Unit<br>Royal Brisbane and Women's Hospital<br>Level 5, Ned Hanlon Building<br>Butterfield Street<br>HERSTON Queensland 4029<br>T: +61 7 3346 6014<br>F: + 61 7 3636 1769<br>E: p.colditz@uq.edu.au

© 2021 Hunt RW et al. *JAMA Network Open*.

Professor Karen Simmer<br>Director, Neonatology Clinical Care Unit (NCCU)<br>King Edward Memorial and Princess Margaret Hospitals<br>1st Floor, Block A<br>374 Bagot Road<br>SUBIACO Western Australia 6008<br>T: +61 8 9340 1262<br>F: +61 8 9340 1266<br>E: Karen.simmer@health.wa.gov.au	Dr Koert de Waal<br>Neonatologist<br>Neonatal Intensive Care Unit<br>John Hunter Children's Hospital<br>Lookout Road<br>NEW LAMBTON New South Wales 2310<br>T: +61 2 49214362<br>F: +61 2 4921 4408<br>E: Koert.DeWaal@hnehealth.nsw.gov.au
Dr Bevan Headley<br>Neonatologist<br>Department of Neonatal Medicine<br>Women's and Children's Hospital<br>72 King William Road<br>NORTH ADELAIDE South Australia 5006<br>T: +61 8 8161 7631<br>F: +61 8 8161 7654<br>E: Bevan.Headley@health.sa.gov.au	Associate Professor David Osborn<br>Royal Prince Alfred Newborn Care<br>Royal Prince Alfred Hospital<br>Missenden Road<br>CAMPERDOWN New South Wales 2050<br>T: +61 2 6515 8363<br>F: +61 2 9550 4375<br>E: david.osborn@email.cs.nsw.gov.au
Associate Professor Peter Dargaville<br>Director, Neonatal and Paediatric Intensive Care Unit<br>Royal Hobart Hospital<br>48 Liverpool Street<br>HOBART Tasmania 7000<br>T: +61 3 6222 7546<br>F: + 61 3 6226 4864<br>E: peter.dargaville@dhhs.tas.gov.au	Dr Alison Tigg<br>Consultant Paediatrician<br>Cairns Hospital<br>The Esplanade<br>CAIRNS Queensland 4870<br>T: +61 7 4226 6659<br>F: +61 7 4226 6714<br>E: Alison.Tigg@health.qld.gov.au
Associate Professor V S Rajadurai<br>Senior Consultant and Head<br>Department of Neonatology<br>KK Women's and Children's Hospital<br>100 Bukit Timah Road<br>SINGAPORE 229899<br>T: +65 6394 1228<br>F: +65 6291 9079<br>E: Victor.Samuel@kkh.com.sg	Dr Monika Olischar<br>Neonatologist<br>University Children's Hospital Vienna<br>Wahringer Gurtel 18-20<br>1090 VIENNA Austria<br>T: +43 1 40400 2147<br>E: monika.olischar@meduniwien.ac.at

1.2.2 Statistician

Dr Katherine Lee PhD MSc
Biostatistician
Clinical Epidemiololgy and Biostatistics Unit
Murdoch Children's Research Unit
Flemington Road
PARKVILLE Victoria 3052

© 2021 Hunt RW et al. *JAMA Network Open*.

T: +61 3 9345 6549
F: +61 3 9345 6000
E: Katherine.lee@mcri.edu.au

1.2.3 Internal Trial Committees

Trial Steering Committee – Dr Rod Hunt (Chair), Professor Paul Colditz, A/Prof Terrie Inder, Dr Nadia Badawi, Dr Jeanie Cheong, Dr Helen Liley, Professor Karen Simmer, Dr Ian Wright, Dr David Osborn (See Appendix 2: Terms of Reference)

1.2.4 Independent Data and Safety Monitoring Committee

(DSMC) – Professor Brian Darlow, Professor Heather Jeffery, Dr Julie Simpson, Professor Janet Rennie.

1.3 Funding and resources

This study is fully funded through a Project Grant from the National Health and Medical Research Council.

2. INTRODUCTION AND BACKGROUND

2.1 Background Information

Neonatal seizures have historically been *defined* as paroxysmal alterations in neurological function, which have behavioural, motor or autonomic manifestations, and which occur in the first 28 days after birth[1]. They are the most distinctive sign of neonatal encephalopathy[2]. However more recently it is realized that many seizures in the newborn period are sub-clinical, and are only identified electrographically[3]. Electrographically documented seizures with or without clinical manifestations is now the more accurately viewed concept of neonatal seizures. Seizures occur more commonly in the neonatal period than at any other time of life and at this developmental stage they are the most frequent manifestation of neurological injury or abnormality[4]. Clinical neonatal seizures occur with a *frequency 1.8 to 3.5 per 1000 term live births*[5]. Seizure identification may be crucial as a marker of neuro-pathology that might otherwise go unrecognised until later childhood.

Outcomes following neonatal seizures are poor. Mortality is reported in up to 20% of term infants who have seizures[6]. With regards to long term morbidity, neonatal seizures have been strongly associated with post-neonatal epilepsy[7], particularly in children with cerebral palsy where the odds of developing epilepsy following neonatal seizures is reportedly 12.9, (95%CI 4.64 – 36.43), $p<0.001$[8]. Neonatal seizures have also been associated with permanent impairments in learning, memory and cognition in up to 47% of survivors[9,10,11,12].

The *aetiology* of seizures is multifactorial. Most reports suggest that the commonest cause of neonatal seizures is hypoxic-ischaemic encephalopathy (HIE), although local epidemiological studies suggest that intrapartum asphyxia only accounts for 29% of newborn encephalopathy[13]. Other causes of newborn seizures include

© 2021 Hunt RW et al. *JAMA Network Open*.

arterial ischaemic stroke[14], sino-venous thrombosis[15], infection, birth-related subarachnoid and subdural haemorrhage and rarely cerebral dysgenesis, metabolic and rare genetic causes[5]. Some clinicians believe that outcome is entirely dictated by aetiology, leading to some ambivalence about the need to detect or treat seizures. Until recently detection of electrographic seizures relied upon conventional electroencephalography (cEEG), an investigation that was generally unavailable at the time of clinical concern, was rarely available outside of routine working hours and required interpretation by paediatric neurologists.

Many clinicians now believe that recurrent seizures are *harmful to the developing brain*[16]. However there is also evidence emerging that synaptic plasticity is altered after a single neonatal seizure[17]. In the rat pup, seizures induced by kainic acid, superimposed onto a hypoxic-ischaemic injury resulted in significantly more neuronal damage than occurred in pups with hypoxic-ischaemic injury alone[18]. In the newborn piglet experimental model of hypoxia-ischaemia, the presence of seizures was associated with increased brain injury detected *in-vivo* by diffusion-weighted magnetic resonance imaging (DW-MRI) and magnetic resonance spectroscopy (MRS). At postmortem the animals that had seized had the greatest degree of neuropathological injury, with cortex, basal ganglia and hippocampi being most vulnerable[19].

Recognition of seizures in the newborn is difficult with identification of only 50%, regardless of experience of the assessor[20]. It is increasingly reported that the majority of neonatal seizures occur sub-clinically, with only 19% of total seizure burden having a clinical manifestation and only 9% of electrographic seizures being identified clinically by neonatal staff[21].

Amplitude-integrated EEG (aEEG) is a bedside tool that provides 2 channel EEG monitoring in real time over a prolonged period[22, 23]. The EEG signal is recorded from superficially placed electrodes (corresponding to P3 and P4, and C3 and C4 according to the international EEG 10-20 classification). Approximately 80% of all neonatal seizures appear in the C3 to C4 channel[24]. Most newborn intensive care units (NICUs) have acquired this monitoring tool, and clinical decisions are sometimes based on the detection of seizures using this instrument[22,23]. Early reports questioned its sensitivity for the detection of neonatal seizures[24]. However, a study comparing aEEG with simultaneously obtained cEEG found that the combination of aEEG and real time 2 channel raw EEG had sensitivity and specificity of 76% and 78% respectively for the detection of electrographic seizures. The majority of electrographic seizures in at-risk newborn infants were detected using aEEG[25]. aEEG monitoring has been further refined by the development of a seizure detection algorithm based on wave-sequence analysis[26]. This algorithm has sensitivity of 83-95% and specificity of 87-94% for the detection of seizures in the neonatal population. In a small randomised, controlled trial of treatment of sub-clinical seizures detected with aEEG compared with treatment of clinical seizures alone, total seizure burden was reduced in infants where electrographic seizures were actively managed[27]. Bedside monitoring of cerebral electrical activity is becoming commonplace in NICU's around Australia and the rest of the world. It is therefore

© 2021 Hunt RW et al. *JAMA Network Open*.

crucial that we investigate the therapeutic role of these monitoring devices before equipoise is lost and we embark upon ‘standard care’ that is not based on evidence.

Management of neonatal seizures has not changed significantly in the last 20 years, despite increasing evidence of the ineffectiveness of current therapy, and the availability of a range of new antiepileptic drugs. First-line therapy for neonatal seizures is phenobarbitone, usually given at a loading dose of 20 mg/kg. The approach to the management of neonatal seizures is consistent across disciplines, with paediatric neurologists and neonatologists choosing phenobarbitone as first-line more than 90% of the time, in both North America[28] and Australia[29]. In many newborn intensive care units (NICU’s) around Australia clinicians now opt for a benzodiazepine as a second- or third-line anticonvulsant[30]. There are also reports emerging of the off-label use of newer anti-convulsant, despite no evidence of safety in neonates or pharmacokinetic data being available for these agents in the newborn, and no randomised trial evidence to support their use[31]. There are reports from animal experiments that exposure of the developing brain to anti-convulsant medication in large doses can result in increased neuronal apoptosis and modify brain development[32].

Summary: *Neonatal seizures are an important sign of neurological disease. There is evidence emerging that neonatal seizures may themselves cause harm. It is also acknowledged that clinical seizures in newborn infants are difficult to recognise and that electrographic seizures may have the same potential for harm, even without a clinical correlate. Many NICU’s are now investing in bedside aEEG monitoring although the evidence for improved clinical outcome following use of such monitoring is limited. This multicentre randomized controlled trial will therefore address the question of whether or not treating electrographic seizures detected by aEEG results in improved neuro-developmental outcome at two years of age.*

2.2 Research Question

Does the treatment of both electrographic and clinical seizures result in reduced mortality and improved long-term outcome relative to treatment of clinical seizures alone?

2.3 Rationale for Current Study

This study is being conducted at this time because of the explosion in use of bedside aEEG monitors in NICU’s around Australia and abroad. Whilst this technology offers the opportunity to detect electrographic seizures over a prolonged period, we lack evidence that the treatment of these electrical events results in improved (or worsened) outcome. It is the intention of this study to answer this primary question.

3. STUDY OBJECTIVES

3.1 Primary Objective

To determine if the active management of electrographic and clinical seizures in encephalopathic term or near-term infants improves mortality and severe disability at two years of age, compared to when only clinically detected seizures are treated.

© 2021 Hunt RW et al. *JAMA Network Open*.

3.2 Secondary Objectives

1. To compare the effect of treatment of EEG seizures detected on aEEG monitoring, on brain injury assessed by cerebral magnetic resonance imaging (MRI) performed at day 5 to 14 compared to when only clinically detected seizures are treated.
2. To explore correlations between seizure burden and the extent of cerebral injury assessed by MRI at day 5 to 14 in term or near term encephalopathic infants.
3. To compare clinically meaningful short term outcomes such as time to full suck feeds and length of hospital stay between the two groups.

4. STUDY DESIGN

4.1 Type of Study

- Prospective randomized, parallel controlled trial.
- treatment groups – intervention and active control (receiving standard neonatal care)
- Hospital-based multi-centre, inpatient subjects. Follow-up at 2 years as outpatients.
- Designed to determine superiority.

4.2 Study Design Diagram

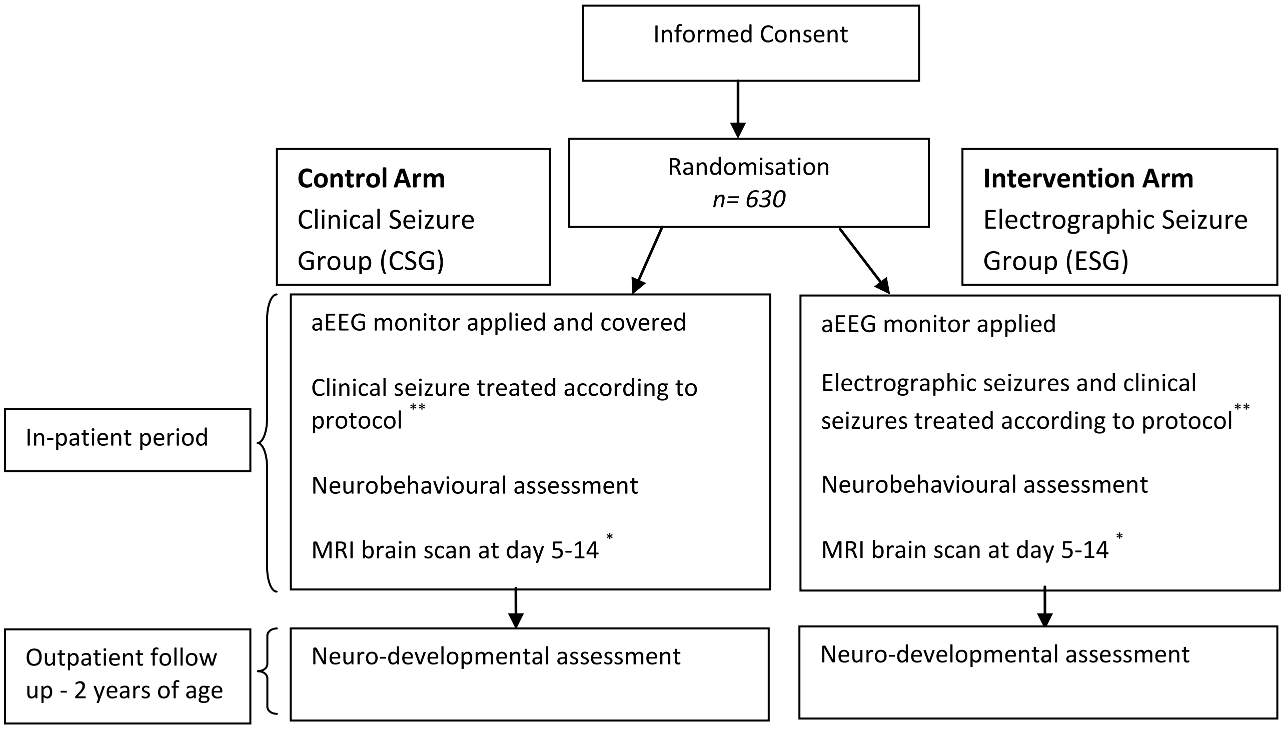

** Conventional *EEG* will be *taken as soon as possible to determine convulsive state. Infants in non-convulsive status epilepticus will be deemed to be a screening failure and will be withdrawn from the study.*

* *All infants will have an MRI brain scan taken at day 5-14. We anticipate some infants will have been discharged home by this time and will therefore be asked to return to the hospital for the scan to be taken. Infants identified with cerebral dysgenesis will be deemed to be a screening failure and withdrawn from the study.*

© 2021 Hunt RW et al. *JAMA Network Open*.

4.3 Number of Subjects

630 – enrolment of 70% of eligible participants attending the study sites over three years of recruitment.

4.4 Expected Duration of Study

3 years of recruitment of subjects and 2 years of follow-up. There will then be a period of six months of data cleaning and analysis with publication of results expected to begin in 2017.

4.5 Primary and Secondary Outcome Measures

Primary Outcome: – comparison of death and severe disability at 2 years of age between treatment and control groups. Severe disability is defined as a cognitive score or composite language score (whichever is worse) > 2SD below the mean, blindness or cerebral palsy with gross motor function classification system [GMFCS] 4 or 5. The medical assessment conducted at 2 years of age will include a neurological assessment at which presence and severity of Cerebral Palsy (CP) can be assessed.

Secondary Outcomes include:

1) MRI detected injury at day 5 to 14 day - detection will be by a combination of qualitative scoring and quantitative assessment of diffusion measures in pre-specified cerebral regions using the scoring system utilized in other studies that Dr Hunt has been involved in where Magnetic Resonance injury is scored (see Appendix 4).
2) Seizure burden - defined as cumulative time seizing in the seven days following recruitment to the study, calculated from aEEG as the time raw EEG showed seizure activity. If seizures resolve and clinical improvement allows removal of the monitor earlier than day 7, then seizure burden will be calculated for the duration the monitor is applied.
3) Anticonvulsant use - both inpatient and outpatient
   - will be measured as:
   1. Number of anticonvulsant drugs used and
   2. Cumulative dose expressed in milligrams/kilogram for each anticonvulsant used
4) Time to full suck feeds
5) Time to discharge from hospital
6) Development of epilepsy by the age of 2 years

5. STUDY TREATMENTS

5.1 Treatment Arms

5.1.1 Description

Eligible infants will be randomised to either have electrographic and clinical seizures treated (as detected by continuous aEEG) or only have clinical seizures treated (with the aEEG monitor in place but the screen covered or the display modified).

5.2 Measurement of subject compliance

Enrolled infants will all be managed according to the study algorithm. Deviations from the algorithm will be recorded (see Appendix 1).

© 2021 Hunt RW et al. *JAMA Network Open*.

5.3 Excluded medications and treatments

Pharmacological management of seizures will be by study protocol (Appendix 1) unless infants require more than three anticonvulsants, at which point therapy will be at the discretion of the treating clinician. We do not expect exclusions based on additional treatments being administered.

6. SUBJECT ENROLLMENT AND RANDOMISATION

6.1 Recruitment

Eligible infants will be identified on admission to the newborn intensive care unit at one of the participating sites. Identification of eligible subjects will be performed by the admitting medical attendant, with delegated responsibility to a Research Nurse or Neonatal Consultant, Fellow or Registrar. Potential participants will be identified as any infant admitted with encephalopathy of any cause and admitted at less than 48 hours of age.

Once admitted, parents of eligible infants will be approached to determine if they would consider participation in a research study. If they indicate willingness in this regard, they will be provided with a parent information sheet (approved by local HREC). They will then be given time to read the document and then discuss the study with a delegated team member. Questions will be asked and answered to determine whether the parent is able to provide informed consent. Consent will then be obtained in writing, photocopied and a copy provided to the parent.

Telephone consent may be sought from the parent/guardian of unaccompanied potential participants who are approaching 48 hours of age. The approach will be conducted by one of the research team and the study telephone consent script will be read to the parent/guardian. A record of verbal consent will be completed by the person obtaining the consent to record the telephone consent process. Parents/guardians will be followed up upon arrival at the hospital by study personnel and written consent obtained. Parents/guardians who provide verbal consent will receive a copy of both consent forms.

6.2 Eligibility Criteria

6.2.1 Inclusion Criteria

- Infants ≥ 35 weeks' gestation (term or near term) at birth admitted to the neonatal intensive care unit
- ≤ 48 hours old at the time of randomization
- A diagnosis of either:
  - **Neonatal encephalopathy** including coma, stupor, or depressed mental state (based on modified Sarnat classification II-III[33])
  - **Hypoxic-ischemic encephalopathy** or at risk for hypoxic-ischemic encephalopathy (i.e. 2 of the following)
    - Apgar score < 5 at 5 minutes
    - Cord blood gas or postnatal blood gas within 1 hour of birth with pH <7.1 or BE > –12 within one hour of birth
    - Need for ongoing respiratory support at 10 minutes after birth

© 2021 Hunt RW et al. *JAMA Network Open*.

- **Suspected neonatal seizures** with an assessment of whether the infant is truly having seizures being made on arrival at the NICU by the treating physician. If the infant is thought to have had seizures, they will be deemed eligible for the trial.

6.2.2 Exclusion Criteria

- Infants < 35 completed weeks of gestation (term or near-term) at birth
- Infants more than 48 hours old at the time of randomization
- Diagnosis of non-convulsive status epilepticus
- Cerebral dysgenesis

6.3 Randomisation Procedures

Randomisation will be stratified by study site and diagnosis (Hypoxic-Ischemic encephalopathy or other). The treatment allocation will be computer generated using block randomisation with variable block sizes by an independent statistician in the Clinical Epidemiology and Biostatistics Unit (CEBU) at The Royal Children's Hospital.

Randomisation will be web-based and available to all centres 24 hours a day. To randomise a participant, researchers will log into the designated site via the link https://eresearch.mcri.edu.au/Logon.aspx?ReturnUrl=%2fprojects%2fCebuRandomisation%2fDefault.aspx using the log in details provided by the central coordinating centre at the site initiation visit. The researcher will choose the NEST study from the drop down list and complete the "New Participant details " – Screening number, date of birth, diagnosis. Once this information has been entered, researchers will click the "Proceed" button to receive the allocation for that participant. Notification of the allocation will be sent via email to the researcher and the Study Coordinator.

In the event of the website being inaccessible, researchers will be able to contact either the CPI (Dr Rod Hunt) or the Study Co-ordinator (Samantha Francis-Pester) by calling the study mobile telephone and a manual randomisation using opaque envelopes will be performed. Site investigators will be provided with this mobile telephone number and will be asked to make contact in the case of website failure so that manual randomisation can proceed.

6.4 Blinding Arrangements

It is not possible to fully blind this study due to the nature of the intervention. Treating doctors and nurses will know which infants are randomised to the aEEG arm and which are randomised to the arm where the monitor is covered or the screen is modified.

However, assessment of the primary outcome will be by a psychologist who was not involved with the family or subject at the time of recruitment and acute illness. Parents will be asked not to divulge which arm of the study their infant was in (where this is known by them). The psychologist assessing the primary outcome will thus be blinded.

6.5 Subject Withdrawal

6.5.1 Reasons for withdrawal

© 2021 Hunt RW et al. *JAMA Network Open*.

Infants can be withdrawn at any stage at the parent's discretion. They will not need to provide a reason for withdrawal. These infants will receive standard care at the treating hospital. Participant's who choose to withdraw will be asked for permission to use already collected data, if further data can be collected and if they can still attend for follow up.

Following randomisation, there are two points at which infants might be withdrawn from this study due to ineligibility. The first is within a few days of randomisation when a conventional EEG is obtained. Where this shows non-convulsive status epilepticus, the infant will be withdrawn so that anti-convulsant treatment can be escalated at the clinician's discretion. At day 5 to 14, the participants will have an MRI brain scan, and where this shows a congenital structural brain abnormality (cerebral dysgenesis), the infant will be withdrawn from the study. The withdrawal of infants on either ground will not compromise the validity of the research data as participants will have been randomly allocated and will be withdrawn irrespective of treatment arm negating any bias.

6.5.2 Handling of withdrawals and losses to follow-up

Towards the end of the second year of recruitment, an assessment will be made of the number of withdrawals and these subjects will be replaced one for one with ongoing recruitment until the target recruitment is reached.

Measures will be employed to maintain the patient cohort and thereby minimise losses to follow up. Contact details for both parents and an additional relative or contact will be collected at enrolment and a birthday card will be sent to all participants on their first birthday. A stamped reply envelope will be included with all birthday cards to allow feedback regarding any changes to the participants contact details. A study newsletter will also be sent to all families at six monthly intervals to maintain communication.

We do not anticipate that the losses to follow-up at the two year assessment will exceed 10% of recruits, based on loss to follow-up rates in many other longitudinal studies recruiting newborn infants in Victoria. We have therefore calculated a sample size that will allow a meaningful result to be obtained at this loss to follow-up rate.

6.5.3 Replacements

Withdrawals will be replaced as per 6.4.2.

6.6 Trial Closure

A Data and Safety Monitoring Committee (DSMC) will be formed as per 1.2.4. Data will be reviewed when recruitment targets of 50, 150, 300 and 450 of 630 participants have been achieved. Data to be reviewed will include assessment of mortality for both arms of the trial. If a difference in mortality in the two arms of the trial satisfies the study DSMC's stopping rule, this information will be relayed to the Trial Steering Committee with an appropriate recommendation (refer to Appendix 3 for DSMC Terms of Reference).

© 2021 Hunt RW et al. *JAMA Network Open*.

7. STUDY VISITS AND PROCEDURES SCHEDULE

STUDY PERIOD	Admission	Inpatient period	Day 7	Day 5 -14	Discharge	2 Year follow up (+/- 2 months)
Informed Consent	X					
Demographic Information	X					
Neurological examination- Sarnat stage	X					
Medical History	X					
Confirm eligibility	X	X		X		
Randomisation		X				
aEEG Monitor attached		X				
aEEG Monitor removed			X[a]			
cEEG		X				
Seizure burden		X		X		
Anticonvulsant drug use		X		X		X
MRI				X		
Dubowitz			X[b]			
Time to full oral feeds					X	
Length of hospital stay					X	
Adverse event check		X		X	X	X
Bayley SID III						X
Post-neonatal epilepsy rate						X

[a] aEEG may be removed prior to day 7 if clinical improvement allow this – refer to Page 18

[b] Dubowitz may be performed prior to day 7 if discharge is prior to day 7.

© 2021 Hunt RW et al. *JAMA Network Open*.

8. CLINICAL AND LABORATORY ASSESSMENTS

- Amplitude-integrated EEG (aEEG). Interpretation of the aEEG underpins the difference between the intervention and the control (standard treatment) arms of this trial. The principal investigators at each of the participating sites have extensive experience reading aEEG at the bedside, but generally acknowledge that whilst they are confident they can recognise sub-clinical seizures on the monitor, they are unsure whether or not to treat them, hence the urgent need for this trial.

  Clinicians will be required to interpret the aEEG for treatment purposes. In order to standardise interpretation of aEEG across sites the following measures will be taken:

  - All study personnel will participate in a one hour training session, either at an investigators meeting, or at their site, conducted by Dr Rod Hunt. In this session, education about pattern recognition, seizure detection, and recognition of false positive patterns (eg. due to biological and environmental artefacts) will be provided. A quiz at the end of the session will require a pass rate of 80% or more in order for personnel to be accredited for study participation.

  - The only atlas of aEEG will be provided to each participating centre prior to the commencement of recruitment at the centre (Hellstrom-Westas L, DeVries LS and Rosen I. Atlas of Amplitude-Integrated EEG's in the Newborn, 2nd Edition, 2008; Informa Health Care: ISBN-13: 978 1 84184 649 1)

  - 10% of all aEEG's will be reviewed off-line by a panel of experts including Dr Rod Hunt, Professor Terrie Inder and Dr Michael Hayman (Paediatric neurologist). This review will be conducted six monthly, and feedback provided to site investigators about the accuracy of their aEEG interpretation.

  - All aEEG Monitors used in this study will be upgraded with Recognize® software. This software was developed in association with the product development by Brainz Instruments, and will show an easily visible flashing alarm when a seizure pattern is displayed on the monitor. With 1:1 nursing of these patients, this alarm will be quickly detected by bedside staff. The seizure detection algorithm that forms the Recognize® software has been shown to have sensitivity for seizure detection ranging from 83 to 95% and positive predictive value of 48-77%.[34] These values are not ideal, but represent a significant improvement over clinical detection of seizures, which is the current standard of care. Whilst this software will not be solely relied upon for seizure detection, it provides a fail-safe for seizures that are not recognised by trained bedside clinical staff or study investigators, and provides an opportunity for review by approved study personnel.

© 2021 Hunt RW et al. *JAMA Network Open*.

- An aEEG monitor will be attached to all participants for seven days or until clinical improvement allows the infant to be transferred from an intensive care to an open cot, for establishment of enteral feeding and preparation for discharge to home.

- Conventional EEG- performed for all participants to confirm participant is not in status epilepticus. cEEG's will be performed as soon as practicable following enrolment depending on availability of this investigation at individual sites. Trial participants will be able to have further cEEG's at any time at the request of the treating clinician. Most centres involved in this trial will be perinatal centres where access to a neurology service providing cEEG is very limited.

- Cerebral MRI at day 5 to 14 – this time frame is deliberate to allow different centres to obtain imaging within this time period. This MRI will not require anaesthesia or sedation. Infants will be fed, wrapped in a vacu-fix® beanbag and imaged whilst asleep. Sequences obtained will include conventional T1- and T2-weighted imaging, as well as diffusion weighted imaging and magnetic resonance spectroscopy. The exact parameters for these sequences will vary from centre to centre depending on the make and strength of the magnet. Advice will be provided by the co-ordinating study centre to ensure that comparable images are obtained from all sites.

- Dubowitz examination - at day 7. This is a standard neurobehavioural assessment performed by either the site investigator or research nurse and takes approximately 10 to 15 minutes[35].

- Standard neuropsychological assessment - at two years of age. This will be the Bayley Scales of Infant Development, 3rd Edition (BSID-III). This will involve a single hospital visit lasting approximately 2 hours. Participant's families will be reimbursed for travel expenses as per section 12.5 of this protocol.

9. ADVERSE EVENT REPORTING

Adverse event (see 9.1) data will be collected on all participants and will be reported at the principal investigators discretion. Any adverse event considered to be due to the natural progression of the disease will not be reported to the DMC or HREC.

9.1 Definitions

9.1.1 Adverse event (AE) is any untoward medical occurrence in a patient enrolled into a study regardless of its causal relationship to study treatment.

9.1.2 Serious adverse event (SAE) is defined as any untoward medical occurrence that:

- Results in death;
- Is life-threatening
- Requires inpatient hospitalisation or prolongation of existing hospitalisation
- Results in persistent or significant disability/incapacity;

© 2021 Hunt RW et al. *JAMA Network Open*.

- Is a congenital anomaly/birth defect
- A medically important event or reaction.

NOTE: The term 'life-threatening' in this definition of 'serious' refers to an event in which the patient was at risk of death at the time of the event; it does not refer to an event which hypothetically might have caused death if it were more severe.

9.1.3 Serious unexpected suspected adverse reactions (SUSAR) is defined as any serious adverse event for which there is some degree of probability that the event is an adverse reaction to the intervention and the adverse reaction is unexpected.

9.2 Assessment and Documentation of Adverse Events

Adverse events attributable to the natural progression of the disease will be recorded on the CRF but not reported to the DMC or Human Research Ethics Committee (HREC).

Anticonvulsants are used in this study as per standard clinical practice. Only those reactions not expected from information obtained from scientific literature or product information and are assessed as an SAE or SUSAR by the site PI or CPI will be reported to the local HREC and DMC.

Medical judgement will be used by both the treating clinician and the site investigators to assess adverse events whilst the participants are in-patients. All adverse events occurring during the in-patient period will be followed up until resolution; until the condition stabilises; until the event is otherwise explained or until the participant is lost to follow-up. Once resolved, the appropriate CRF and the adverse event log will be updated.

No adverse event data will be recorded beyond the initial inpatient period, until the two-year follow-up, when parents will be asked if they have observed anything of concern that might relate to the study.

The investigator will make an assessment of intensity for each SAE reported during the study and will be recorded on the CRF. The assessment will be based on the investigator's clinical judgement. The intensity of each SAE recorded in the CRF should be assigned to one of the following categories:

- **Mild:** An event that is easily tolerated by the subject, causing minimal discomfort and not interfering with everyday activities
- **Moderate:** An event that is sufficiently discomforting to interfere with normal everyday activities.
- **Severe:** An event which is incapacitating and prevents normal everyday activities.

Severity is a category utilised for rating the intensity of an event. An event is defined as "serious" when it meets one of the predefined outcomes as described in Section 9.1 "Definition of an SAE".

© 2021 Hunt RW et al. *JAMA Network Open*.

Assessment of Causality
The investigator will assess the relationship between the intervention and the occurrence of each SAE and their assessment will be recorded on the CRF. The investigator will use clinical judgment to determine the relationship. Alternative causes, such as natural history of the underlying diseases, concomitant therapy, other risk factors, and the temporal relationship of the event to the intervention will be considered and investigated. The causal relationship to the intervention assessed by the Investigator (or medically qualified delegate) will be assessed using the following classifications:

- **Not Related** In the Investigator's opinion, there is not a causal relationship between the study treatment and the adverse event.

- **Unlikely** The temporal association between the adverse event and intervention is such that the study treatment is not likely to have any reasonable association with the adverse event.

- **Possible** The adverse event could have been caused by the study participant's clinical state or the treatment.

- **Probable** The adverse event follows a reasonable temporal sequence from the commencement of study treatment and cannot be reasonably explained by the known characteristics of the participant's clinical state.

- **Definitely** The adverse event follows a reasonable temporal sequence from the time of study treatment

9.3 Eliciting Adverse Event Information

Participants in this study will be monitored daily by the site investigator or employed research assistant/nurse whilst an inpatient. Any clinical event thought to relate to the study will be reported to the site investigator, or the Principal Investigator, and a decision made as to whether the event was part of the natural progression of the disease or whether it was related to study procedure or lack thereof.

For each adverse event, start and stop dates, action taken, outcome, intensity (see above) and causality will be documented. If an AE changes in frequency or intensity during the study, a new entry of the event will be made in the CRF.

9.4 Serious Adverse Event Reporting

9.4.1 SAEs

SAE'S occurring in participants where they are thought to relate in any way to the study procedure or lack thereof, will be reported to the local HREC within 48 hours of occurrence using the local reporting system. SAE follow up will be the responsibility of the site PI.

SAE's will be recorded on the CRF and the adverse event log.

As death is a primary outcome of this research, any death or serious adverse event attributable to the natural progression of the disease will be recorded on the CRF but not reported to the DMC or HREC.

© 2021 Hunt RW et al. *JAMA Network Open*.

9.4.2 SUSARs

SAE's considered to be a SUSAR by the site PI or CPI will be reported to the local HREC within 24 hours unless otherwise specified by the local HREC.

10. STATISTICAL METHODS

10.1 Sample Size Estimation

Our meta-analysis of therapeutic hypothermia trials found that 49% of infants with moderate or severe HIE who were cooled either died or had severe neurodisability at 2 years[42]. Cooling is now standard care for infants with moderate or severe HIE. Only about half of subjects recruited to this study will have seizures because of HIE – many others will seize secondary to other aetiologies, where outcome may not be as bad e.g. stroke, subarachnoid bleeding secondary to birth trauma. We therefore estimate a background risk of death or neurodisability in our cohort of about 40%. In the meta-analysis of therapeutic hypothermia, major disability in survivors was reduced from 42 to 29% [42]. There is no data on what effect could be obtained with the use of electrographic as well as clinical monitoring of seizures however we could plausibly expect a similar effect as seen in the cooling studies.

Thus we base our power calculation on finding a 12% reduction in death or severe disability which would be an extremely important difference in practice. Recruitment of 260 infants in each group will be required to detect a 12% reduction in death or severe disability at 2 years (alpha value of 0.05 and power of 80%). As indicated above, some infants initially eligible will be excluded post-randomisation because ineligibilities will be identified during the study. Allowing for a post-randomisation exclusion rate of 5% plus a loss to follow-up rate of 10%, 299 infants will be recruited to each group.

The following number of eligible subjects per annum are expected at each centre based on the number of term encephalopathic infants presenting to each centre per annum over the past 3 years; Royal Children's Hospital, Melbourne = 25; Royal Women's Hospital, Melbourne = 30; Children's Hospital at Westmead, Sydney = 20; Westmead Hospital = 25; Mater Mothers' Hospital, Brisbane = 20; Royal Brisbane and Women's = 15; Perth (King Edward Memorial Hospital and Princess Margaret Hospital) = 40; Newcastle = 25; Adelaide = 20; Royal Prince Alfred Hospital = 15; Royal Hobart Hospital = 5; St Louis NICU, St Louis, Missouri, USA = 25.

Based on these numbers we predict that in all participating centres 795 infants will be eligible in the 3 year study period. Enrolment of 79% of these infants will be necessary to complete recruitment within 3 years.

10.2 Population to be analysed

Statistical analysis will be performed according to intention to treat where outcome data are available. If the aEEG monitor fails, the infant will still be included in the treatment arm, but a protocol violation will be recorded and information about all violations will be fed back to the Data Monitoring Committee.

© 2021 Hunt RW et al. *JAMA Network Open*.

10.3 Statistical Analysis Plan

The primary outcome is death or neurodisability at 2 years of age. This will be summarised as the number and proportion in each intervention group. Comparisons between the groups will be tested using a chi-squared test, with the difference between the groups presented as a difference in proportions and its 95% confidence interval.

Secondary outcomes will also be summarised by intervention group, presented as numbers and proportions for categorical outcomes and means and standard deviations for continuous outcomes (or medians and inter-quartile ranges for non-normal data). Comparisons between the groups will be made using t-tests with differences presented as mean differences and 95% confidence intervals (or Mann-Whitney tests for non-normal data) and chi-squared tests, with the difference presented as a difference in proportions and its 95% confidence interval. Time to event outcomes will be analysed on the log scale. If there are censored data for these outcomes, survival analysis may be considered.

All results will be presented as unadjusted comparisons however results will also be adjusted for site and diagnosis (stratification factors), and whether the infant was cooled (for HIE infants) using linear and logistic regression.

10.4 Interim Analyses

Interim analyses will be carried out by the study statistician as per the DMC Terms of Reference and presented to the DMC to assess mortality differences between the two arms of the study. The number and proportion of infants who have died will be presented by treatment arm, along with difference in proportions and its 95% confidence interval. There will be no other interim analysis until all data is collected.

11. DATA MANAGEMENT

11.1 Data Collection

Study nurses and local investigators will manually record data onto a paper CRF from the participant's medical record. CRF's will be de-identified, identifiable only by a study specific unique identifier. A list linking participants to study numbers will be kept in a locked cabinet at each site.

The CRF will be photocopied. The copy will be stored at the recruiting centre and the original will be posted to the co-ordinating site as follows:

Ms Samantha Francis-Pester
Neonatal Research
Level 4, West Building
Murdoch Childrens Research Institute
The Royal Children's Hospital
50 Flemington Road
PARKVILLEVIC 3052

© 2021 Hunt RW et al. *JAMA Network Open*.

Data will then be entered into an electronic database at The Royal Children's Hospital. This database will be accessible by Dr Rod Hunt and the Study Co-ordinator and will be password protected. The database will be backed up to a portable hard-drive weekly – this will also be password protected.

CRF's will be retained in participant files and stored in a locked cabinet during the study. Upon study closure all CRF's will be archived and retained for 25 years.

MRI data will be de-identified and then copied onto CD and sent to the Co-ordinating site (address as above).

Quality control: CPI and Study Co-ordinator will be conducting regular site visits to all participating centres (on average annual visit) for monitoring purposes.

11.2 Data Storage

Hard copy data on paper CRF's will be accessible by CPI and Study Co-ordinator and stored in a locked filing cabinet in the CPI's locked office.
At site level, we would encourage site investigators to store their copy of the paper CRF in a locked cabinet with limited access.

11.3 Study Record Retention

All study related documents will be stored until the 25th birthday of the youngest participant of the study. Data will be stored in a secure location with limited access at each participating site and will be the responsibility of the site PI. All paper data will then be securely shredded and electronic data destroyed.

12. ADMINISTRATIVE ASPECTS

12.1 Confidentiality

Subject confidentiality is strictly held in trust by the participating investigators, research staff, the sponsoring institution and their agents. The study protocol, documentation, data and all other information generated will be held in strict confidence. No information concerning the study or the data will be released to any unauthorised third party, without prior written approval of the sponsoring institution. Authorised representatives of the sponsoring institution may inspect all documents and records required to be maintained by the Investigator, including but not limited to, hospital medical records and pharmacy records for the participants in this study. The clinical study site will permit access to such records. All records and reports that leave the site will be identified only by the SID number to maintain participant confidentiality. Clinical information will not be released without written permission of the participant, except as necessary for monitoring by an HREC or regulatory agencies.

© 2021 Hunt RW et al. *JAMA Network Open*.

12.2 Independent HREC Approval

This protocol and the informed consent document and any subsequent modifications will be reviewed and approved by an independent HREC. A letter of protocol approval from the HREC will be obtained prior to the commencement of the study, as well as approval for other study documents subject to HREC review.

12.3 Modifications of the protocol

This study will be conducted in compliance with the current version of the protocol. Any change to the protocol document or Informed Consent Form that affects the scientific intent, study design, patient safety or may affect a participants willingness to continue participation in the study is considered an amendment, and therefore will be written and filed as an amendment to this protocol and/or Informed Consent Form. All such amendments will be submitted to the HREC's, for approval prior to becoming effective.

12.4 Protocol Deviations

Protocol deviations will be minimised by education at each participating site prior to local study commencement. Additional education of study team members will be conducted as necessary throughout the study should issues with protocol compliance be identified.

All protocol deviations will be recorded in the patient record and on the CRF and will be reported to the PI. Protocol deviations will be assessed for significance by the PI. Those deviations deemed to have a potential impact on the integrity of the study data, patient safety or ethical acceptability of the study will be reported to the HREC in a timely manner.

Where deviations to the protocol identify issues for protocol review, the protocol will be amended as per section 12.3.

12.5 Participant Reimbursement

Expenses related to the day 5 -14 MRI and two year follow-up, such as parking, petrol to maximum of $20 or public transport costs, will be reimbursed upon presentation of receipts. A claim form and self-addressed envelope will be provided to parents/guardians to facilitate the reimbursement of costs incurred.

12.6 Financial Disclosure and Conflicts of Interest

There are no existing financial disclosures or conflicts of interest.

All researchers are obligated to disclose any conflicts of interest that may arise during the conduct of the study. Remedial strategies will include independent monitoring of the study, modification of the researcher's responsibilities and exclusion of the researcher from continued participation in all or a portion of the study.

© 2021 Hunt RW et al. *JAMA Network Open*.

13. USE OF DATA AND PUBLICATIONS POLICY

The CPI will hold primary responsibility for publication of the results of the study, and any use of the data for other studies. Approval will need to be sought and obtained in writing from the CPI before any information can be used or passed on to a third party.

It is the intention of the CPI to ensure that primary outcomes from this study are reported at national and international conferences, and submitted for publication within six months of completion of the trial. Authorship will be offered to any of the site investigators where there is collective agreement that contribution warrants authorship.

© 2021 Hunt RW et al. *JAMA Network Open*.

14. REFERENCES

1. Volpe JJ. Neurology of the Newborn, 5th Edition, 2005. WB Saunders Company
2. Lombroso CT, Holmes GL. Value of the EEG in neonatal seizures. Journal of Epilepsy 1993; 6: 39-70.
3. Bourez-Swart MD, van Rooij L, Rizzo C, deVries LS, Toet MC, Gebbink TA, Ezendam AGJ and van Huffelen AC. Detection of subclinical electroencephalographic seizure patterns with multichannel amplitude-integrated EEG in full-term neonates. Clinical neurophysiology 2009; 120: 1916-22.
4. Mizrahi EM. Acute and chronic effects of seizures in the developing brain: lessons from clinical experience. Epilepsia 1999; 40: S42-50.
5. Silverstein FS and Jensen FE. Neonatal Seizures. Ann Neurol 2007; 62: 112-20.
6. Ronen GM, Buckley D, Penney S, Streiner DL. Long-term prognosis in children with neonatal seizures: a population-based study. Neurology 2007; 69: 1816-22.
7. Clancy RR and Legido A. Postnatal epilepsy after EEG-confirmed neonatal seizures. Epilepsia 1991; 32: 69-76.
8. Zelnik N, Konopnicki M, Bennett-Back O, Castel-Deutsch T and Tirosh E. Risk factors for epilepsy in children with cerebral palsy. European Journal of Paediatric Neurology 2010; 14: 67-72.
9. Sayin U. Sutula TP and Stafstrom CE. Seizures in the developing brain cause adverse long-term effects on spatial learning and anxiety. Epilepsia 2004; 45: 1539-48.
10. McBride MC, Laroia N and Guillet R. Electrographic seizures in neonates correlate with poor neurodevelopmental outcome. Neurology 2000; 55: 506-13.
11. Tekgul H, Gauvreau K, Soul J et al. The current etiologic profile and neurodevelopmental outcome of seizures in term newborn infants. Pediatrics 2006; 117: 1270-80.
12. Thibeault-Eybalin MP, Lortie A and Carmant L. Neonatal Seizures: Do they damage the brain? Pediatr Neurol 2009; 40: 175-80.
13. Badawi N, Kurinczuk JJ, Keogh JM, Alessandri LM, O'Sullivan F, Burton PR, Pemberton PJ and Stanley FJ. Intrapartum risk-factors for newborn encephalopathy: the Western Australian case-control study. BMJ 1998; 317: 1554-8.
14. Hunt RW and Inder TE. Perinatal and Neonatal Ischaemic Stroke: A review. Thrombosis Research 2006; 118: 39-48.
15. Hunt RW, Badawi N, Laing S and Lam A. Pre-eclampsia: A predisposing factor for neonatal venous sinus thrombosis? Paediatric Neurology, 2001; 25: 242-6.
16. Wasterlain CG. Recurrent seizures in the developing brain are harmful. Epilepsia 1997; 38: 728-34.
17. Cornejo BJ, Mesches MH, Coultrap S, Browning MD and Benke TA. A single episode of neonatal seizures permanently alters glutamatergic synapses. Ann Neurol 2007; 61: 411-26.
18. Wirrell EC, Armstrong EA, Osman LD and Yager JY. Prolonged seizures exacerbate perinatal hypoxic-ischemic brain damage. Pediatr Res 2001; 50: 445-54.
19. Bjorkman ST, Miller SM, Rose SE, Burke C and Colditz PB. Seizures are associated with brain injury severity in a neonatal model of hypoxia-ischaemia. Neuroscience 2010; 166: 157-67.
20. Malone A, Ryan CA, Fitzgerald A, Burgoyne L, Connolly S and Boylan GB. Interobserver agreement in neonatal seizure identification. Epilepsia 2009; 50: 2097-101.
21. Murray DM, Boylan GM, Ali I, Ryan CA, Murphy BP and Connolly S. Defining the gap between electrographic seizure burden, clinical expression and staff recognition of neonatal seizures. Arch Dis Child Fetal Neonatal Ed 2008; 93: F187-91.
22. Toet MC and Lemmers PMA. Brain monitoring in neonates. Early Human Development 2009; 85: 77-84.

© 2021 Hunt RW et al. *JAMA Network Open*.

23. Hellstrom Westas L, Rosen I, deVries LS and Greisen G. Amplitude integrated EEG. Classification and interpretation in pre-term and term infants. NeoReviews 2006; 7: e76-87.
24. Shellhass RA, Soaita AI and Clancy RR. Sensitivity of amplitude-integrated electroencephalography for neonatal seizure detection. Pediatrics 2007; 120: 770-7.
25. Shah DK, Mackay MT, Lavery S, Watson S, Harvey AS, Zempel J, Mathur A and Inder TE. Accuracy of bedside electroencephalographic monitoring in comparison with simultaneous continuous conventional electroencephalography for seizure detection in term infants. Pediatrics 2008; 121: 1146-54.
26. Navakatikyan MA, Colditz PB, Burke CJ, Inder TE, Richmond J and Williams CE. Seizure detection algorithm for neonates based on wave-sequence analysis. Clinical neurophysiology 2006; 117: 1190-203.
27. Van Rooij LGM, Toet MC, van Huffelen AC, Groenendaal F, Lann W, Zecic A, de Haan T, van Straaten ILM, Vrancken S, van Wezel G, van der Sluijs J, ter Horst H, Gavilanes D, Laroche S, Naulaers G and de Vries LS. Effect of treatment of subclinical neonatal seizures detected with aEEG: randomized, controlled trial. Pediatrics 2010; 125: e358-66.
28. Blume HK, Garrison MM and Christakis DA. Neonatal Seizures: Treatment and treatment variability in 31 United States Pediatric Hospitals. Journal of Child Neurology 2009; 24: 148-54.
29. Browning Carmo K and Barr P. Drug treatment of neonatal seizures by neonatologists and paediatric neurologists. J Paed Child Health 2005; 41:313-6.
30. Shany E, Benzaqen O and Watemberg N. Comparison of continuous drip of midazolam or lidocaine in the treatment of intractable neonatal seizures. Journal of Child Neurol 2007; 22: 255-9.
31. Sivlerstein FS and Ferriero DM. Off-label use of antiepileptic drugs for the treatment of neonatal seizures. Pediatr Neurol 2008; 39:77-9.
32. Bittigau P, Sifringer M, Genz K, Reith E, Pospischil D, Govindarajalu S, Dzietko M, Pesditschek S, Mai I, Dikranian K, Olney JW and Ikonomidou C. Antiepileptic drugs and apoptotic neurodegeneration in the developing brain. PNAS 2002; 99: 15089-94.
33. Sarnat H and Sarnat M. Neonatal encephalopathy following fetal distress. Archives of Neurology 1976; 33: 696-705.
34. Navakatikyan MA, Colditz PB, Burke CJ, Inder TE, Richmond J and Williams CE. Seizure detection algorithm for neonates based on wave-sequence analysis. Clinical Neurophysiology 2006; 117: 1190-1203.
35. Dubowitz L et al, Mental Retardation and Development 2005; 11: 52-60

© 2021 Hunt RW et al. *JAMA Network Open*.

15. APPENDICES

APPENDIX 1

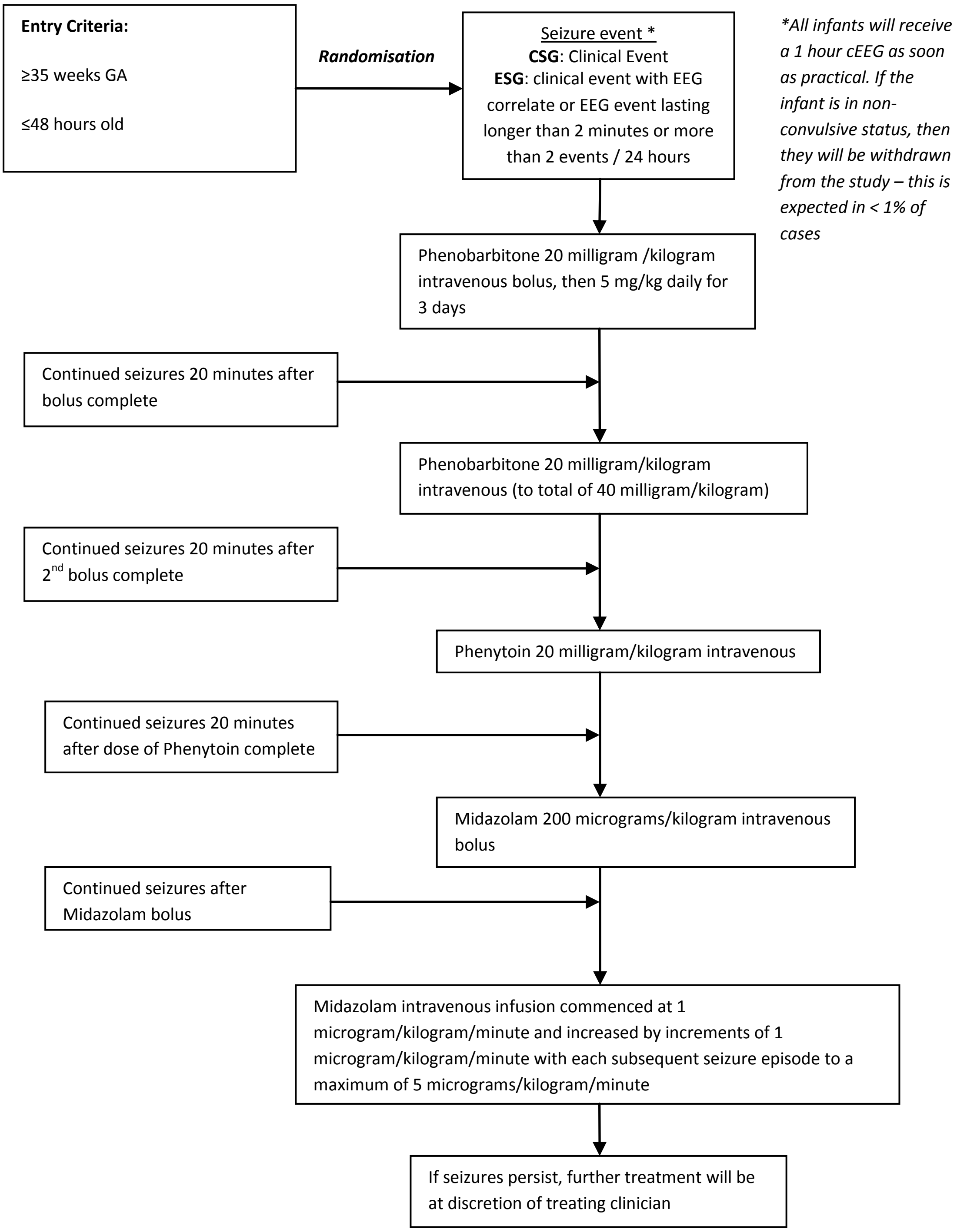

© 2021 Hunt RW et al. *JAMA Network Open*.

Appendix 2

TRIAL STEERING COMMITTEE – TERMS OF REFERENCE

Neonatal Electrographic Seizure Trial

ACTRN: ACTRN12611000327987

Effective Date	
Version	1
Author/Reviewer	Samantha Francis-Pester
Authoriser	Dr Rod Hunt
Review date	

1. Objectives

The Trial Steering Committee (TSC) will be responsible for the scientific integrity of the trial including the validity of the trial protocol, data quality and trial conduct. The TSC will also be responsible for the scientific quality of any publications derived from the use of trial data. The primary objectives of the TSC are to:

- Supervise the overall conduct and progress of the trial, ensuring the trial conduct complies with Good Clinical Practice guidelines and all regulatory requirements
- Ensure adherence to the approved protocol
- Monitor all safety data and new information
- Consider any recommendations of the Data Monitoring Committee or HREC
- Provide an avenue of participant grievance resolution
- Oversee the dissemination of trial results

2. Accountability and Reporting

2.1 Accountability

The TSC will report to the Human Research and Ethics Committee (HREC) and the National Health and Medical Research Council (NHMRC) as the sole provider of trial funding. The TSC will report to the HREC and NHMRC through the TSC Chairperson.

2.2 Reporting

2.2.1 The Trial Coordinator will provide the TSC with the following information:

- Safety reports including all Serious Adverse Event (SAE) and Sudden Unexpected Suspected Adverse Reaction (SUSAR) reports from participating sites

© 2021 Hunt RW et al. *JAMA Network Open*.

- Recruitment figures
- Protocol violation and deviation reports
- Complaint details

Additional information will be provided to the TSC at the discretion of the Coordinating Principal Investigator and the Trial Coordinator. The TSC chairperson may request additional information for consideration at the scheduled TSC meeting by providing a written request to the trial coordinator no later than 2 weeks prior to the meeting date.

2.2.2 Information will be prepared for the immediate reporting period or as specified by written directive from the TSC Chairperson.

2.2.3 The collated information shall be provided to the TSC members no later than two weeks prior to the scheduled date of the TSC meeting.

2.2.4 The TSC will report back to the HREC in a timely manner through the submission of annual reports during the trial period and a final report at trial closure. NHMRC reporting will be annual according to the terms of acceptance of an NHMRC Project Grant.

Reports generated following TSC meetings will be the responsibility of the TSC chairperson and will be provided to HREC and NHMRC according to organizational requirements. A copy will be lodged with the trial coordinator at The Royal Children's Hospital, Melbourne. The delivery of this document will be the responsibility of the author and remains the property of the co-ordinating centre located at The Royal Children's Hospital.

3. Membership of the Trial Steering Committee

3.1 Composition

Membership of the TSC will be limited to 15 members and will include principal investigators from each participating site. The Trial Co-ordinator, statistician and other team members may attend at the request of the TSC or as appropriate.
The TSC will comprise of:

Members

- Dr Rodney Hunt (Vic) - Chair
- Dr Ian Wright (NSW)
- Professor Nadia Badawi (NSW)
- Professor Paul Colditz (QLD)
- Professor Karen Simmer (WA)
-

© 2021 Hunt RW et al. *JAMA Network Open*.

Voting Members

- Trial Statistician – Katherine Lee

Non-Voting members

- Study Co-ordinator – Samantha Francis-Pester

3.2 Role of TSC members

Chairperson – The Chairperson should have previous experience conducting meetings in a manner to facilitate discussion and interaction between attending members. The Chairperson is responsible for the reporting of TSC activity to the HREC and NHMRC.

3.3 Appointment of TSC members

All members will be eligible according to their individual area of expertise, experience in clinical research conduct according to Good Clinical Practice Guidelines and regulatory requirements and/or membership of the trial team.

3.4 Tenure

TSC members will be appointed for the duration of the study.

3.5 Remuneration

Remuneration will not be provided to any member of the TSC.

4. Meetings

4.1 Frequency of meetings

The TSC will convene prior to the commencement of participant recruitment then annually thereafter. Meeting dates will be scheduled for no later than 6 weeks after the reporting period as passed.

All meetings will follow a standardized format and it will be the responsibility of the trial Coordinating Principal Investigator (CPI) – Dr Rod Hunt - to schedule and organize the meeting of the TSC.

The TSC Chairperson or CPI has the option to call additional meetings of the TSC if deemed necessary.

4.2 Meeting agenda

Meeting agendas will be distributed by the Trial Co-ordinator – Samantha Francis-Pester - to all TSC members, at least one week prior to the scheduled meeting date.

4.3 Quorum

A quorum shall consist of 3 members and the Chair.

4.4 Confidentiality

All TSC members must take all reasonable measures to maintain the integrity and credibility of the research project by treating as, and keeping confidential all information and documents relating to this research project.

© 2021 Hunt RW et al. *JAMA Network Open*.

4.5 Records

All records of the TSC will be prepared and maintained by the trial co-ordinator. This includes but is not limited to all agendas, minutes and reports. The TSC Chairperson and the CPI will sign off the meeting minutes prior to distribution.

5. Method of Operation

5.1 Procedures

TSC meetings will be face-to-face meetings of all TSC members and will be closed to all persons except the TSC members, those individuals providing secretariat services and invited guests. The TSC will consider all information provided to the Committee and will deliver clear determinations on each matter.

The CPI and/or Trial Co-ordinator will guide the TSC through the information provided for review.

5.1.1 Decision Making

All decisions made by the TSC will be majority and in circumstances disputation remains, the final decision will rest with the TSC chair.

Possible recommendations can include:

- No action needed, study to continue as planned
- Protocol modification and/or changes to Information statement
- Extending recruitment based on actual accrual rates

5.1.2 Disagreement

Where there is disagreement between the recommendations of the TSC and the DMC, a joint meeting of the membership will be convened to resolve the dispute. The meeting will be chaired by an appointed person with relevant experience in meetings and clinical research conduct who is completely independent of the research team.

© 2021 Hunt RW et al. *JAMA Network Open*.

Appendix 3

DATA AND SAFETY MONITORING COMMITTEE – TERMS OF REFERENCE

Neonatal Electrographic Seizures Trial

ACTRN: ACTRN12611000327987

Effective Date	
Version	1
Author/Reviewer	Samantha Francis-Pester
Authoriser	Dr Rod Hunt
Review date	

1. Objectives

The Data Safety Monitoring Board will be responsible for the review of accruing trial data including updated recruitment data, primary outcomes and safety data.
The primary objectives of the DMC are to:

- Monitor evidence for treatment differences in the main efficacy outcome measures
- Monitor evidence of treatment harm through the review of safety data
- Determine the continued ethical conduct of the study
- Monitor recruitment figures

2. Accountability and Reporting

2.1 Accountability

The DMC will be accountable to the Trial Steering Committee (TSC) and shall report to the TSC through the DMC chairperson.

2.2 Reporting

2.2.1 The DMC will be provided with trial data and safety reports compiled by the trial statistician. Trial data to be provided to the DMC will include but not be limited to:

- data on all-cause death rates, seizure burden and anticonvulsant use blinded by treatment arm (unless unblinded data is requested by DMC members in writing to the trial statistician no less than four weeks prior to the scheduled meeting)
- a detailed summary of all SAE'S and SUSAR'S only
- Recruitment data – total number, recruitment by site, by diagnosis
- Protocol violations and deviations

2.2.2 The data provided to the DMC will be for the immediate reporting period as specified by the study protocol or written directive from the DMC Chairperson.

2.2.3 The data shall be provided to the DMC members no later than two weeks prior to the scheduled date of the DMC meeting.

© 2021 Hunt RW et al. *JAMA Network Open*.

2.2.4 The DMC will report back to the TSC in a timely manner following each meeting by providing written details of any identified issues and/or recommendations.

The letter will be the responsibility of the DMC Chairperson and will be provided to the TSC in hard copy via regular mail and an electronic copy sent via email to the TSC Chairperson. A copy will be lodged with the trial co-ordinator at The Royal Children's Hospital, Melbourne. The delivery of this document will be the responsibility of the author and remains the property of the co-ordinating centre located at The Royal Children's Hospital, Melbourne.

3. Membership of the Data Monitoring Committee

3.1 Composition

All DMC members must be familiar with the study protocol and should have sighted the document prior to agreeing to join the DMC.

Membership of the DMC will be limited to 4 members who include a least one clinician experienced in Neonatology and a statistician. All members of the DMC must be independent of the research team and must not have any vested interest in the research outcomes. Members should be supportive of the aims and methods of the study.

The DMC will comprise of:

- Professor Brian A Darlow — Chair
MA MB BChir MD (Cantab) FRCP FRCPCH
University of Otago
- Professor Heather Jeffery — MMed (Int Pub Health), PhD
University of Sydney
- Dr Julie Simpson — Sc Math & Stat., MSc Stat., PhD
University of Melbourne
- Dr Janet M Rennie — B,ChB, MRCP, FRCP, DCH, MD, FRCPCH
University College London Hospital

3.2 Role of DMC members

Chairperson – The Chairperson should have previous experience of DMC meetings, expertise in both clinical and statistical issues and experience conducting meetings in a manner to facilitate discussion and interaction between attending members. The Chairperson will be responsible for reporting DMC activity to the TSC.
DMC statistician – to provide independent statistical expertise;

Neonatologist – to interpret statistical findings in light of clinical context.

3.3 Appointment of DMC members

All members of the DMC will be eligible according to their individual area of expertise and experience in clinical research conduct according to Good Clinical Practice guidelines and regulatory requirements.

3.4 Tenure

DMC members will be appointed for the duration of the study.

3.5 Remuneration

Remuneration will not be provided to any member of the DMC however costs incurred to attend DMC meetings and/or trial meetings (as requested by the TSC) will be reimbursed.

© 2021 Hunt RW et al. *JAMA Network Open*.

4. Meetings

4.1 Frequency of meetings

The DMC will convene following the achievement of the following recruitment targets:

- 50 participants
- 150 participants
- 300 participants

Meeting dates will be scheduled for no later than 6 weeks after the reporting period has passed.

The CPI and study coordinator should be available to attend the open sessions of the DMC meetings. Other team members will not usually be expected to attend but any team member may attend the open session of a DMC meeting with prior written notification. The DMC may request an individual to attend the open session of the meeting with all requests being made in writing no less than two weeks prior to the scheduled meeting date.

All meetings will follow a standardized format:

- An open session during which a general overview of study status and conduct and any new relevant external data will be given by the CPI and/or Study Coordinator.
- A closed session to all except the trial statistician (or her designee) and DMC membership when the trial statistician (or designee) will present and explain the presented data;
- A closed session to all individuals except the DMC membership

It will be the responsibility of the CPI–Dr Rod Hunt - to schedule and organize the meeting of the DMC.

The DMC chair has the option to call additional meetings of the DMC and request any additional data if deemed to be necessary.

4.2 Meeting agenda

Meeting agendas will be distributed by the Study Co-ordinator, Samantha Francis-Pester, to all DMC members, no less than one week prior to the scheduled meeting date.

4.3 Quorum

A quorum shall consist of 3 members of the DMC.

4.4 Conflict of Interest

Where personal or professional circumstances change, any member of the DMC who believes a real or potential conflict of interest exists with their continued membership of the DMC, the member shall declare this conflict as soon as practicable. Action taken from this declaration will be at the discretion of the other DMC members and shall be binding. This disclosure and any decision made must be minuted.

4.5 Confidentiality

All DMC members must take all reasonable measures to maintain the integrity and credibility of the research project by treating as, and keeping confidential all information and documents relating to this research project.

4.6 Records

All records of the DMC will be prepared and maintained by the Study Co-ordinator. This includes, but is not limited to all agendas and reports. Minutes for open and closed sessions

© 2021 Hunt RW et al. *JAMA Network Open*.

will be prepared by the DMC secretariat and separated for confidentiality purposes. The DMC chair will sign off the minutes and any DMC issued documentation.

5. Method of Operation

5.1 Procedures

DMC meetings will be face-to-face or teleconference meetings and will be comprised of an open and 2-part closed session. The meeting will commence with an open session in which the CPI and/or study coordinator will provide details of the study status and conduct to date. The CPI and/or study coordinator will then be excused and the trial statistician or his/her designee will present interim study data. The trial statistician or designee will guide the DMC members through the data and participate in DMC discussion.

At the conclusion of the first closed session, the meeting will be closed to all persons except the DMC membership and those individuals providing secretariat services. The DMC will consider the data provided and deliver clear recommendations to the TSC.

5.1.1 Decision Making

All decisions made by the DMC will be majority. Every effort should be made to achieve a unanimous decision. Where unanimity cannot be achieved, the final decision will rest with the DMC chair.

Possible recommendations can include:

- No action needed, study to continue as planned
- Early study cessation due to clear benefit or harm of one arm of the study
- Extending recruitment based on actual accrual rates
- Sanctioning and/or proposal of protocol modification.

5.1.2 Stopping Rules

Stopping rules shall be regarded as guidelines rather than rules. The DMC shall not recommend early cessation of the study unless the DMC's review of the safety data and/or rate of death indicates a statistically significant difference with 95% confidence interval of benefit or harm between the study arms. Cessation of the study will not occur without the consultation and agreement of the TSC.

5.1.3 Disagreement

Where there is disagreement between the recommendations of the DMC and the TSC, a joint meeting of the membership will be convened to resolve the dispute. The meeting will be chaired by an appointed person with relevant experience in meeting and clinical research conduct who is completely independent of the research team.

6. Publications

Following the completion of the trial, the Chair of the DMC will be provided with a draft of any publication of trial results to allow review of the role of the DMC reported in the publication and to verify the publication of trial results as per regulatory requirements.

© 2021 Hunt RW et al. *JAMA Network Open*.

Appendix 4

MRI Scoring System

SLICES

Number	Sequence	Level
1	T2 sagittal	midline
2	T2 parasagittal	lateral ventricle
3	T1 or T2 parasagittal slice	sylvian fissure
4	coronal T2 parallel to the axis of the brain stem	anterior part of the frontal lobes
5	coronal T2	third ventricle
6	coronal T2	lateral ventricles' temporal horns
7	coronal T2 parallel to the axis of the brain stem	ventricular atria
8	T1 axial slice perpendicular / axis of the brain stem	fourth ventricle
9	T1 axial slice	third ventricle
10	T2 axial slide	vertex

White Matter

Cystic abnormalities

None =1 *1 region = 2* *2 regions = 3* *3 or more regions = 4*

Score cystic	

Cumulative score

White matter signal

None =1 *1-2 spots = 2* *3-5 spots = 3* *> 5 spots = 4*

	Score	Localisation
Focal T1		

© 2021 Hunt RW et al. *JAMA Network Open*.

Focal T2		

Cumulative score [ ]

None =1 *1 region = 2* *2 regions = 3* *3 or more regions = 4*

Diffuse T2		

Cumulative score [ ]

White matter atropy

None =1 *1 region = 2* *2 regions = 3* *3 or more regions = 4*

White matter atrophy	

Cumulative score [ ]

Subventricular zone
Absent = 1 *Present = 2* *Very prominent = 3*

Score	

Cumulative score [ ]

Ventriculomegaly

Normal (< 10) =1 *Mild (10-15 mm) = 2* *Severe (>15) = 3*

	Left	Right
Atrial diameter		

Cumulative score [ ]

DTI

Normal =1 *? = 2* *? = 3* *? = 4*

		Left	Right
PLIC	ADC		
	RA		
Centrum semi ovale			

© 2021 Hunt RW et al. *JAMA Network Open*.

Frontal	ADC		
	RA		
Central	ADC		
	RA		
Occipital	ADC		
	RA		

Cumulative score ☐

Corpus callosum

normal, with thick corpus callosum visible in all views = 1 focal thinning in the corpus callosum often visible in the mid region of the body of the corpus on sagittal images. = 2 global thinning across the entire corpus callosum = 3

Length (sagittal view)	
Score	

Cumulative score ☐

Myelination
Present = 1 Impaired = 2 Absent = 3

Vermis / middle cerebellar peduncle	
PLIC	

Cumulative score ☐

Normal score = 28

© 2021 Hunt RW et al. *JAMA Network Open*.

Hemorrhage

Intra ventricular
Absent=1 Minimal < 10 % = 2 Moderate 10-50 % = 3 Severe > 50 % = 4

	Left	Right
IVH		

Cerebellum
Absent = 1 present < 50 %= 2 > 50 % = 3

	Left	Right
Cerebellum hemorrhage		

Parenchymal hemorrhage
None =1 1 region = 2 2 regions = 3 3 or more regions = 4

	Left	Right
Parenchymal hemorrhage		

Cumulative score

Normal score = 6

Grey matter

Deep nuclear grey matter
Normal = 1 mild = 2 severe = 3

	Left	Right
Signal		
Atrophy		

Hippocampus
Normal = 1 mild = 2 severe = 3

© 2021 Hunt RW et al. *JAMA Network Open*.

	Left	Right
Hippocampus		

Cumulative score

Normal score = 6

Gyration

	Absent	Present	*< 30 weeks*	*30 weeks*	*34 weeks*	*36-40 weeks*
Precentral gyrus			+	+	+	+
Post-central gyrus			+	+	+	+
Superior frontal sulcus				+	+	+
Inferior frontal sulcus				+	+	+
Temporal sulci					+	+
Secondary occipital sulci						+
Tertiary occipital sulci						+

Normal for CA = 1 *Delayed 2 weeks = 2*

Delayed 4 weeks= 3 *Delayed 6 weeks = 4*

Score gyration	

Normal score = 1

© 2021 Hunt RW et al. *JAMA Network Open*.

Global Atrophy

Subarachnoidal space

Normal = 1 *mild = 2* *moderate = 3* *severe = 4*

Subarachnoidal space	

Cumulative score ☐

Measurement

Normal = 1 *mild = 2* *moderate = 3* *severe = 4*

Bone biparietal diameter	
Cerebral biparietal diameter	
Ratio Cerebral/Bone	
Interhemispheric distance	
Antero posterior interopecular distance	
Cranio caudal interopercular distance	
Cerebellum transverse diameter	

Cumulative score ☐

Number of coronal slices with CSF before brain

Normal = 1 *mild = 2* *moderate = 3* *severe = 4*

Cumulative score ☐

Normal score = 9

© 2021 Hunt RW et al. *JAMA Network Open*.
