# Supplementary material for: Effect of Treatment of Clinical Seizures vs Electrographic Seizures in Full-Term and Near-Term Neonates: A Randomized Clinical Trial
Source: JAMA Netw Open. 2021 Dec 17;4(12):e2139604. doi: 10.1001/jamanetworkopen.2021.39604 (PMC8683963; doi:10.1001/jamanetworkopen.2021.39604)
Supplement: Supplement 2. — eFigure. Treatment Algorithm for Seizures [file jamanetwopen-e2139604-s002.pdf]

Supplemental Online Content

Hunt RW, Liley HG, Wagh D, et al; Newborn Electrographic Seizure Trial Investigators. Effect of treatment of clinical seizures vs electrographic seizures in full-term and near-term neonates: a randomized clinical trial. *JAMA Netw Open*. 2021;4(12):e2139604. doi:10.1001/jamanetworkopen.2021.39604

eFigure. Treatment Algorithm for Seizures

This supplemental material has been provided by the authors to give readers additional information about their work.

© 2021 Hunt RW et al. *JAMA Network Open.*

eFigure. Treatment Algorithm for Seizures

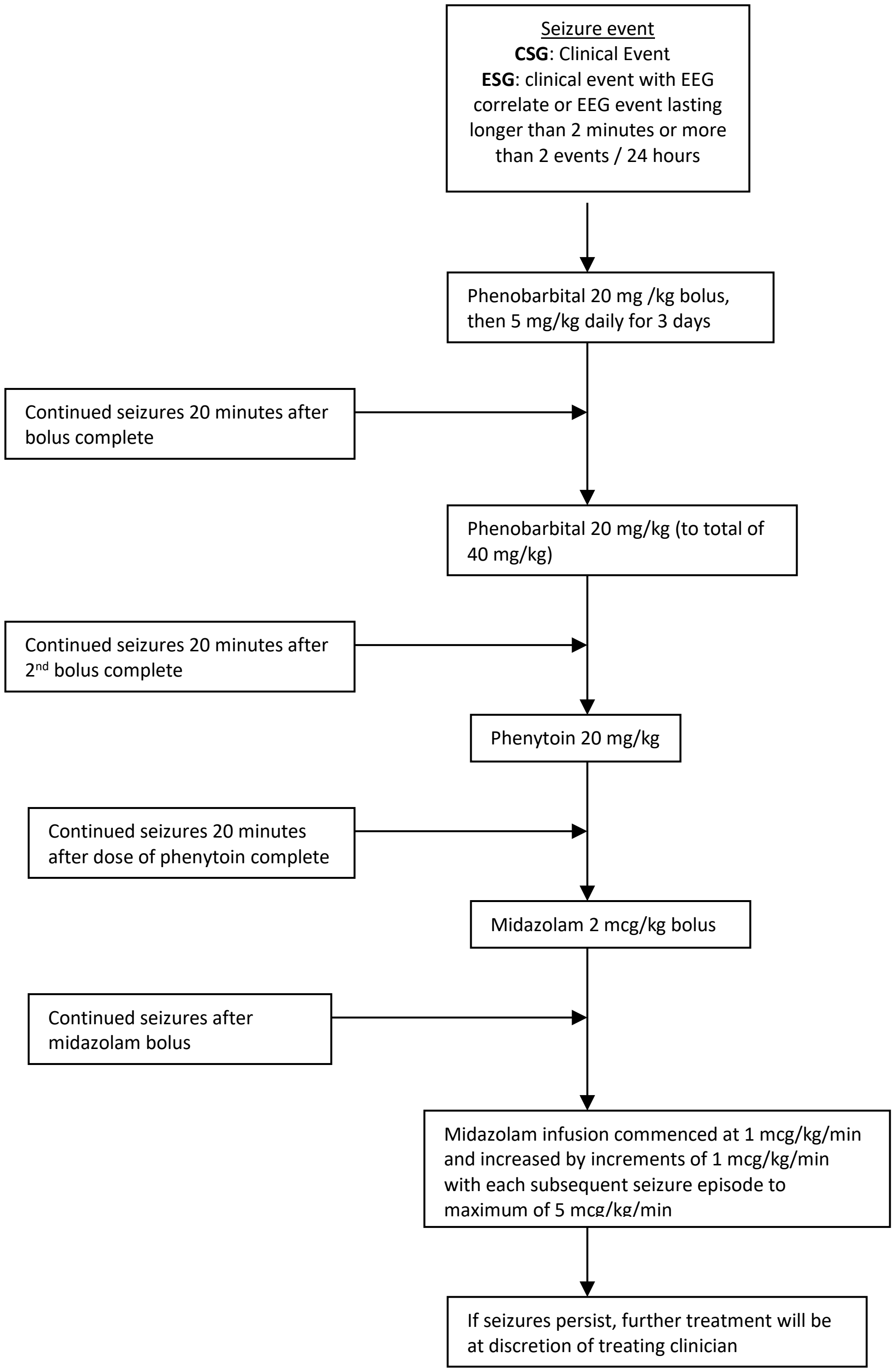

© 2021 Hunt RW et al. *JAMA Network Open.*
