# Supplementary material for: Effect of Treatment of Clinical Seizures vs Electrographic Seizures in Full-Term and Near-Term Neonates: A Randomized Clinical Trial
Source: JAMA Netw Open. 2021 Dec 17;4(12):e2139604. doi: 10.1001/jamanetworkopen.2021.39604 (PMC8683963; doi:10.1001/jamanetworkopen.2021.39604)
Supplement: Supplement 3. — Group Members [file jamanetwopen-e2139604-s003.pdf]

Supplemental Online Content: Nonauthor Collaborators

*Indicates required information. Only first name, last name, and suffix will appear in PubMed.

*Group Name(s): Newborn Electrographic Seizure Trial Investigators							
***First Name and Middle Initial(s)**	***Last Name**	***Suffix (eg, Jr, III)**	Academic Degrees	Institution	Location (city, state/province, country)	Role or Contribution, eg, chair, principal investigator	Group (if more than 1 Group listed in the byline) and/or Subgroup (eg, Steering Committee)
Susan E	Jacobs		MD	The Royal Women's Hospital	Melbourne, Vic, Australia	Site Associate Investigator	
Connie	Wong			The Royal Women's Hospital	Melbourne, Vic, Australia	Research Nurse	
Emma	Yeomans			Monash Children's Hospital	Melbourne, Vic, Australia	Research Nurse	
Kristy	Elsayed			Monash Children's Hospital	Melbourne, Vic, Australia	Research Nurse	
Judith	Macey			Mater Mother's Hospital	Brisbane, Qld, Australia	Research Nurse	
Jan	Stewart			The Children's Hospital	Westmead, NSW, Australia	Research Nurse	
Karen	Simmer		PhD	King Edward Memorial Hospital	Perth, WA, Australia	Site Associate Investigator	
Yen	Kok			King Edward Memorial Hospital	Perth, WA, Australia	Research Nurse	
Ross	Black			John Hunter Children's Hospital	Newcastle, NSW, Australia	Research Nurse	
Lisa	McKeown			Royal Brisbane and Women's Hospital	Brisbane, Qld, Australia	Research Nurse	
Nicky	Roberts			King Edward Memorial Hospital	Perth, WA, Australia	Research Nurse	
Nirmal K	Visruthan			KK Women's and Children's Hospital	Singapore	Research Nurse	
Rowena	Dela Puerta			KK Women's and Children's Hospital	Singapore	Research Nurse	
Stacey	Worthington			King Edward Memorial Hospital	Perth, WA, Australia	Research Nurse	
