# Supplementary material for: Effect of Treatment of Clinical Seizures vs Electrographic Seizures in Full-Term and Near-Term Neonates: A Randomized Clinical Trial
Source: JAMA Netw Open. 2021 Dec 17;4(12):e2139604. doi: 10.1001/jamanetworkopen.2021.39604 (PMC8683963; doi:10.1001/jamanetworkopen.2021.39604)
Supplement: Supplement 4. — Data Sharing Statement [file jamanetwopen-e2139604-s004.pdf]

**Data Sharing Statement**

Hunt RW, Liley HG, Wagh D, et al; Newborn Electrographic Seizure Trial Investigators. Effect of treatment of clinical seizures vs electrographic seizures in full-term and near-term neonates: a randomized clinical trial. *JAMA Netw Open*. 2021;4(12):e2139604. doi:10.1001/jamanetworkopen.2021.39604

**Data**
**Data available:** Yes
**Data types:** Deidentified participant data, Data dictionary
**How to access data:** Deidentified participant data and data request will be made available upon reasonable request to the corresponding author: Professor Rod Hunt
rod.hunt@monash.edu
**When available:** With publication

**Supporting Documents**
**Document types:** None

**Additional Information**
**Who can access the data:** Researchers whose proposed use of the data has been approved.
**Types of analyses:** Systematic review with either meta-analysis or individual patient data analysis
**Mechanisms of data availability:** with investigator support
**Any additional restrictions:** nil
